# Chloride intracellular channel 1 activity is not required for glioblastoma development but its inhibition dictates glioma stem cell responsivity to novel biguanide derivatives

**DOI:** 10.1186/s13046-021-02213-0

**Published:** 2022-02-08

**Authors:** Federica Barbieri, Alessia Graziana Bosio, Alessandra Pattarozzi, Michele Tonelli, Adriana Bajetto, Ivan Verduci, Francesca Cianci, Gaetano Cannavale, Luca M. G. Palloni, Valeria Francesconi, Stefano Thellung, Pietro Fiaschi, Samanta Mazzetti, Silvia Schenone, Beatrice Balboni, Stefania Girotto, Paolo Malatesta, Antonio Daga, Gianluigi Zona, Michele Mazzanti, Tullio Florio

**Affiliations:** 1 Sezione di Farmacologia, Dipartimento di Medicina Interna, Università di Genova, Viale Benedetto Xv, 2, 16132 Genoa, Italy; 2 Dipartimento di Farmacia, Università di Genova, 16132 Genoa, Italy; 3 Dipartimento di Bioscienze, Università degli Studi di Milano, Via Celoria 26, 20133 Milan, Italy; 4 IRCCS, Ospedale Policlinico San Martino, 16132 Genoa, Italy; 5 Dipartimento di Neuroscienze, Riabilitazione, Oftalmologia, Genetica e Scienze Materno-Infantili, Università di Genova, 16132 Genoa, Italy; 6 Fondazione Grigioni per il Morbo di Parkinson, 20135 Milan, Italy; 7 Computational and Chemical Biology, Fondazione Istituto Italiano di Tecnologia, 16163 Genoa, Italy; 8 Department of Pharmacy and Biotechnology, University of Bologna, 40126 Bologna, Italy

**Keywords:** Glioblastoma stem cells, Targeted therapy, CLIC1, Biguanide derivatives, GBM organoids

## Abstract

**Background:**

Chloride intracellular channel-1 (CLIC1) activity controls glioblastoma proliferation. Metformin exerts antitumor effects in glioblastoma stem cells (GSCs) inhibiting CLIC1 activity, but its low potency hampers its translation in clinical settings.

**Methods:**

We synthesized a small library of novel biguanide-based compounds that were tested as antiproliferative agents for GSCs derived from human glioblastomas, in vitro using 2D and 3D cultures and in vivo in the zebrafish model. Compounds were compared to metformin for both potency and efficacy in the inhibition of GSC proliferation in vitro (MTT, Trypan blue exclusion assays, and EdU labeling) and in vivo (zebrafish model), migration (Boyden chamber assay), invasiveness (Matrigel invasion assay), self-renewal (spherogenesis assay), and CLIC1 activity (electrophysiology recordings), as well as for the absence of off-target toxicity (effects on normal stem cells and toxicity for zebrafish and chick embryos).

**Results:**

We identified Q48 and Q54 as two novel CLIC1 blockers, characterized by higher antiproliferative potency than metformin in vitro, in both GSC 2D cultures and 3D spheroids. Q48 and Q54 also impaired GSC self-renewal, migration and invasion, and displayed low systemic in vivo toxicity. Q54 reduced in vivo proliferation of GSCs xenotransplanted in zebrafish hindbrain. Target specificity was confirmed by recombinant CLIC1 binding experiments using microscale thermophoresis approach. Finally, we characterized GSCs from GBMs spontaneously expressing low CLIC1 protein, demonstrating their ability to grow in vivo and to retain stem-like phenotype and functional features in vitro. In these GSCs, Q48 and Q54 displayed reduced potency and efficacy as antiproliferative agents as compared to high CLIC1-expressing tumors. However, in 3D cultures, metformin and Q48 (but not Q54) inhibited proliferation, which was dependent on the inhibition dihydrofolate reductase activity.

**Conclusions:**

These data highlight that, while CLIC1 is dispensable for the development of a subset of glioblastomas*,* it acts as a booster of proliferation in the majority of these tumors and its functional expression is required for biguanide antitumor class-effects. In particular, the biguanide-based derivatives Q48 and Q54, represent the leads to develop novel compounds endowed with better pharmacological profiles than metformin, to act as CLIC1-blockers for the treatment of CLIC1-expressing glioblastomas, in a precision medicine approach.

**Supplementary Information:**

The online version contains supplementary material available at 10.1186/s13046-021-02213-0.

## Background

Chloride intracellular channels (CLIC1–6) are non-canonical self-assembling anion channels involved in physiological cell functions as well as in cancer development [1]. In particular, CLIC1 regulates cell cycle progression, proliferation, migration and apoptosis in several solid tumors [2, 3] including melanoma, colorectal, lung, ovarian, pancreatic, prostate, and breast cancer, [4], and glioblastoma (GBM) [5–7], and was proposed as prognostic biomarker and therapeutic target [3, 8]. For example, CLIC1-expressing pancreatic cancer patients undergo to a worse overall survival as compared with patients developing CLIC1-negative tumors [9]; CLIC1 upregulation is correlated to chemotherapy resistance in ovarian cancer, [10], and with clinical aggressiveness and metastatic diffusion in colorectal cancer [11].

CLIC1 exists as either soluble cytosolic protein or transmembrane isoform (tmCLIC1), assembled in multimeric ion channel conformation [12] and its functional overexpression in cancer cells supports its possible pharmacological targeting as innovative therapeutic option [1].

GBM is a molecularly complex and heterogeneous primary brain tumor exhibiting high lethality despite aggressive multimodal treatment (combination of surgery, radiation and chemotherapy) [13], due to rapid cell growth and brain parenchyma invasion. Currently, all GBM patients receive similar standardized treatment [14], although increasing evidence supports the relevance of specific pharmacogenetic-based targeted treatments for different subgroups of cancer patient to achieve successful results [15, 16]. In this view, GBM molecular subtype classification pushes toward the development of individualized treatments [17, 18]; however, to achieve this goal, the improvement of patient-derived GBM preclinical models is mandatory, including 2D and 3D (organoids) cultures of patient-derived GBM stem cells (GSCs). GSCs are the subpopulation responsible for initiation, progression and recurrence of solid tumors and are characterized by self-renewal, differentiation and tumorigenic abilities and, chemo- and radio-resistance [19–21]. In GBM, CLIC1 upregulation in the active tmCLIC1 conformation [5, 7, 22] correlates with aggressiveness [7] and, in low grade gliomas, with reduced patients’ survival [23]. In human GSCs, inward Cl^−^ current, via tmCLIC1, regulates reactive oxygen species (ROS) accumulation, and causes pH changes to promote cell cycle progression [24]. Notably, CLIC1 activity is crucial for GSC survival, proliferation, and invasiveness [6, 7], showing higher membrane localization and ionotropic activity than in non-stem cell population. Although, the direct contribution of CLIC1 to GSC tumorigenesis has not been completely elucidated, its downregulation by siRNA, inhibits GSC proliferation in vitro [6] and tumorigenesis in vivo [7], suggesting a high therapeutic potential of its pharmacological targeting. Conversely, this protein is less relevant for the survival/proliferation of normal stem cells (i.e. mesenchymal stem cells) and non-stem (differentiated) GBM cells composing the bulk of tumor, in which CLIC1 is mainly cytosolic and displays low activity as ion channel [6].

The improved knowledge of the molecular mechanisms of cancer growth favored the exploitation of drug repurposing, identifying antineoplastic activity of compounds developed for other diseases via a target-driven selection which allows the targeting of new vulnerabilities of cancer cells [25]. Moreover, target selection is required in determining which patients will respond to targeted drugs, directing patient care towards precision medicine approaches.

The prototype of non-oncology drugs repurposed as anticancer agent is metformin, a first-line anti-hyperglycemic drug for type-2 diabetes [26]. Metformin inhibits GSC proliferation in vitro [27] and in vivo [28], as well as GSCs self-renewal, metabolic processes, migration, and angiogenesis [29–32] via both direct and indirect molecular mechanisms, which include: (i) modulation of insulin/insulin-like growth factor-1, mitogen-activated protein kinase, and AMP-activated protein kinase regulated pathways; (ii) inhibition of mitochondrial energy metabolism; (iii) interference with ROS and transforming growth factor-β signaling [33]. However, the relative relevance of all these mechanisms in metformin antitumor activity is still not completely clarified.

In this context, the evidence that metformin inhibition of GSC proliferation and migration is dependent on the blockade of CLIC1-mediated ion currents [6], paved the way for the hypothesis that CLIC1 functional expression determines tumor cell sensitivity to metformin [34].

However, translational studies and retrospective analyses in diabetic metformin users, although confirming safety of the treatment [35], reported contradictory results about its anticancer efficacy [36, 37]. In particular, pharmacokinetic issues and the low potency observed in vitro would require supra-pharmacological doses to obtain clinical effects. Thus, the efficacy of structurally-related derivatives, acting via CLIC1 inhibition with higher potency than metformin, has been analyzed [8]. Besides metformin and the related antidiabetic drug phenformin, other clinically approved (currently or in the past) compounds containing a biguanide core-structure showed antitumor activity, including anti-flu (moroxydine) and anti-malaria (cycloguanil) agents. Importantly, the biguanide moiety was present in these molecules as a linear (metformin, phenformin, moroxydine) or cyclized structure (i.e. cycloguanil), although both molecular families were able to affect GSC proliferation via CLIC1 inhibition, providing the proof-of-concept of a pharmacological class-effect [38]. However, despite improvements in potency, efficacy, or toxicological issues were observed, compounds with a better pharmacological profile than metformin, suitable for further development in clinical studies, have not been identified yet.

In the present study, patient-derived GSCs from different GBMs, characterized for CLIC1 expression, were used as monolayer, 3D organoids, and in vivo after zebrafish xenograft, to investigate the role of CLIC1 in GSC growth. In particular, we have tested the effects of CLIC1 blockade by novel biguanide derivatives focusing on GSC proliferation, migration, and invasion. We highlight the relevance of the selection of tumors which express this biomarker to obtain positive responses to treatments with the novel compounds we developed, and move toward a patient tailored therapy for GBM.

## Methods

### Tumor samples

Glioma post-surgical specimens were obtained from the Neurosurgery Department of IRCCS Ospedale Policlinico San Martino (Genova, Italy) after patients’ informed consent and Institutional Ethical Committee approval (CER Liguria register number 360/2019). All patients underwent surgery for the first time and never received chemo- or radio-therapy. Clinical, histopathological and molecular features of patients and tumors, classified as glioma grade IV (*n* = 8) and grade III (*n* = 1) according to WHO criteria, are reported in Table S1.

### RNA sequencing

RNA was extracted from 10^6 cells with Qiagen RNeasy mini kit following manufacturer’s recommendations. RNA Quality was checked with Agilent TapeStation and ranged between RIN 8.6 to 9.9. Library preparation was carried out with Truseq stranded mRNA (Illumina) and sequenced on Illumina platform with a 150 cycles paired end flowcells. Up to 80 M reads were obtained for each sample. Reads were aligned with STAR 2.7.5c [39] and counted with RSEM 1.3.2 [40] using GRCh38.p13 as reference genome.

### Glioblastoma stem cell (GSC), umbilical cord mesenchymal stem cell (ucMSC), and rat astrocyte primary cultures

Patient-derived GBM primary cell cultures were isolated and characterized as previously detailed [41] and maintained in vitro in serum-free medium containing 1:1 DMEM-F12/Neurobasal™ (EuroClone, Milano, Italy/Gibco-ThermoFisher Scientific, Monza, Italy), B27™ supplement (Gibco-ThermoFisher Scientific), 2 mM L-glutamine (EuroClone), 1% penicillin-streptomycin (EuroClone), 15 μg/ml insulin (Sigma-Aldrich, Milano, Italy), 2 μg/ml heparin (Sigma-Aldrich) and completed with recombinant human bFGF (10 ng/ml; Miltenyi Biotec, Bologna, Italy) and EGF (20 ng/ml; Miltenyi Biotec). Under these conditions cells gave rise to floating tumor-spheres within 2 weeks, but to allow feasible and reliable experiments cells were grown as monolayers in flasks coated with growth factor-reduced Matrigel™ (Corning, ThermoFisher Scientific), ensuring the maintenance of stem cell marker expressions, spherogenic properties, differentiation and tumorigenic potential [41]. Tumor-initiating activity was confirmed by orthotopic xenografts in 6–8-weeks old non-obese diabetic severe combined immunodeficient (NOD/SCID) mice, injecting 10,000 cells (see Table S1).

To induce differentiation, GSCs were shifted for 2 weeks in DMEM-F12 medium supplemented with 10% FBS 2 mM L-glutamine, 1% penicillin-streptomycin (all from EuroClone).

Human umbilical cords (uc, *n* = 4) were collected from full-term women, immediately after caesarean section at the Obstetrics and Gynecology Department (International Evangelical Hospital, Genova, Italy), after informed consent and Institutional Ethic Committee approval (register number 2/2010). Uc-mesenchymal stem cells (ucMSCs) were isolated as reported [42], cultured in MesenPRO-RS™ Medium (Gibco-ThermoFisher Scientific), and fully characterized by flow cytometry (MSC Phenotyping Kit, Miltenyi Biotec) following the criteria of the International Society for Cellular Therapy [43].

Sprague–Dawley rat astrocytes were obtained by 7-day old pup cerebral cortices, isolated according standard procedures [44]. Experimental procedures and animal care complied with the EU Parliament and Council Directive (2010/63/EU) and were approved by the Italian Ministry of Health (prot. 75F11.N.6DX) in accordance with D.M. 116/1992. All efforts were made to minimize animal suffering and to reduce the number of animals used for the experiments.

### CLIC1 gene silencing

Short hairpin mRNA recognizing human CLIC1 (5′-GATGATGAGGAGATCGAGCTC-3′) and firefly luciferase (5′-CGTACGCGGAATACTTCGA-3′) sequences were cloned into the XhoI/HpaI sites of the pLentiLox 3.7 lentiviral vector [7] and stably expressed in GBM19 GSCs.

### Chemicals, reagents, and antibodies

Metformin, methotrexate, 1-phenylbiguanide hydrochloride (Q46) and 1-(4-chlorophenyl)-biguanide hydrochloride (Q42) are commercially available (Sigma-Aldrich). Other linear biguanides (Q48, Q49, Q50) were synthesized by refluxing an ethanolic solution of the proper aniline derivative hydrochloride with dicyandiamide, according to previously published protocols: Q48 and Q49 [45], Q50 [46].

Cycloguanil analogues, Q51, Q52, Q53 [47], and Q54 [48], were achieved by an acid-catalyzed, three-component synthesis involving an aniline derivative, dicyandiamide and acetone. To monitor compounds’ purity, elemental analysis of synthesized compounds was performed on a Flash 2000 CHNS (Thermo Scientific) instrument at the Microanalysis Laboratory of Pharmacy Department (University of Genova); purity > 95% was observed for all the newly synthesized compounds (Table S2).

The following primary antibodies were used:

- Anti-CLIC1 (356.1, SantaCruz Biotechnology, Heidelberg, Germany) dil. 1:750 (WB)
- Anti-Sox2 (sex determining region Y-box 2, L1D6A2, Cell Signaling Technology, EuroClone) dil. 1:100 (IF), 1:1000 (WB)
- Anti-GFAP (glial fibrillary acidic protein, GA5, Cell Signaling Technology, EuroClone) dil. 1:1000 (WB), 1:100 (IF)
- Anti-β-III-Tubulin (18207, Abcam Cambridge, UK) dil. 1:100 (IF)
- Anti-Olig2 (oligodendrocyte transcription factor 2, EPR2673, Abcam) dil. 1:100 (IF)
- Anti-α-tubulin (Sigma-Aldrich) dil. 1:7500 (WB).
- Anti phospho ERK1/2 (Cell Signaling Technology, EuroClone) dil. 1:1000 (WB)

### Immunohistochemistry

Tissue sections were sequentially incubated with: i) 3% H_2_O_2_ for 20 min; ii) 1% BSA diluted in phosphate saline buffer 0.01 M (PBS) containing 0.1% Triton X-100 for 20 min (BPBS); iii) the primary antibody, anti-CLIC1 (in BPBS) overnight, at room temperature (RT). Antigen-antibody binding was visualized using EnVision anti-mouse secondary antibody (1 h at RT, Dako, Milano, Italy) and with 3,3′-Dia-minobenzidine as chromogen (DAB, Dako kit). Tissue sections were counterstained with hematoxylin and mounted with permanent mounting medium (Eukitt). The specificity of the primary antibody was assessed by incubating adjacent sections from the same GBM with BPBS in place of CLIC1 antibody.

### Cell proliferation assays

#### MTT reduction assay

MTT (3-(4,5-dimethylthiazol-2-yl)-2,5-diphenyltetrazolium bromide, Sigma-Aldrich) tetrazolium reduction assay was used to test cell viability. GSCs were plated in 96-well plates at the concentration of 3000 cells/well. After 48 h treatment with test compounds, MTT substrate (2.5 mg/ml, in PBS) was added to cells and incubated for 2 h. The quantity of formazan crystals formed was measured, after being dissolved in DMSO (Sigma-Aldrich), by recording absorbance at 570 nm using a BioTek ELx800 plate reading spectrophotometer [49].

#### Cell count of viable cells

Trypan blue exclusion assay was used to evaluate cell viability reduction induced by test compounds. GSCs (100,000 cells/well) were seeded in 6-well plates, and after 24 h were treated with drugs for further 48-72 h. Viable cells were counted in the presence of Trypan blue 0.4% w/v (Bio-Rad Laboratories, Milano, Italy) using the TC-20™ automated cell counter (Bio-Rad Laboratories) and reported as % of viable cells divided by the total number of counted cells [38].

### Spherogenesis assay

GSCs were seeded in complete medium without Matrigel™, in 48-well plates at 1000 cells/well. After 24 h, cells were exposed to drugs and spherogenesis was monitored for 7 days. The number of spheres/well was quantified using a digital camera mounted on a transmitted light microscope to image each individual well, and visually calculated by two independent operators [50].

### Cell migration and invasion assays

Migration assay was performed using Falcon™ FluoroBlok™ HTS96 transwell Support System (ThermoFisher Scientific) with a light-tight PET membrane with 8 μm pores, that blocks light transmission between 400 and 700 nm. Cells (15,000/well) were labeled with Vybrant™ CFDA-SE Cell Tracer (Invitrogen-ThermoFisher Scientific) [51], plated on inserts, and allowed to migrate towards FBS-containing medium for 18 h. Cells migrated to membrane bottom side, were analyzed by confocal laser-scanning microscope (Bio-Rad MRC 1024-ES) at 10× magnification, and quantified by ImageJ software (NIH, Bethesda, USA).

To test the effects of novel biguanides on GSC invasiveness, cells were seeded without Matrigel™ in complete medium for 1 week to generate spheres. Then, spheres were harvested and embedded in 80% Matrigel™ and 20% complete medium in the presence of Q48 (100 μM), Q54 (100 μM), Q46 (100 μM) and metformin (10 mM). After Matrigel™ polymerization (45 min), complete medium with Q48, Q54, Q46 or metformin was added to the dish (T0) and Matrigel™ invasion was allowed for 15 h (T15). Within this time frame compounds do not impair cell proliferation. Photos were taken using a digital camera ICC50 HD (Leica, Milano, Italy) mounted on a transmitted light microscope DM IL (Leica). Analysis was performed by ImageJ software, calculating at least 2 diameters for each sphere. The differences between the average diameter of T15 and T0 of each condition were calculated and expressed as percentage of vehicle-treated controls.

### Electrophysiology

Patch electrodes (GB150F-8P with filament, Science Products GmbH, Hofheim, Germany) were pulled from hard borosilicate glass on a Brown-Flaming P-87 puller (Sutter Instruments, Novato, CA, United States) and fire-polished to a tip diameter of 1–1.5 μm and an electrical resistance of 5–7 MΩ. Patch-clamp electrophysiology was performed in perforated-patch whole cell configurations, as reported [24]. In patch clamp whole cell experiments the voltage protocol consisted of 800 ms pulses from − 40 mV to + 60 mV every 10 s. In time course trials a 60 mV depolarizing voltage step, 800 ms duration was delivered every 5 s. After 5 min, in which the membrane current was stabilized, we perfused the cell with the test compounds. In both experimental conditions current amplitude was measured as trace average between 700 and 750 ms.

Patch clamp solutions were the following:

- **bath solution** (mM): 140 NaCl, 5 KCl, 10 HEPES, 1 MgCl_2_, 2 CaCl_2_, 5 D-Glucose, pH 7.4;
- **pipette solution** (mM): 135 KCl, 10 HEPES, 10 NaCl, 1 MgCl_2_, 2 CaCl_2_, 5 D-Glucose, pH 7.4.

### Immunofluorescence

GSCs, grown in chamberslides, were fixed with 4% paraformaldehyde (PFA), permeabilized in PBS/0.1% Triton X-100, blocked with normal goat serum and immunostained with primary antibodies, followed by AlexaFluor-568 and -488 fluorochrome-conjugated secondary antibodies (Invitrogen-ThermoFisher Scientific), as reported [52]. Nuclei were counterstained with DAPI (Sigma-Aldrich). Slides were photographed with a DM2500 microscope equipped with a DFC350FX digital camera (Leica).

### Western blotting

Cells were lysed in a buffer containing 1% Igepal, 20 mM Tris–HCl, pH 8, 137 mM NaCl, 10% glycerol, 2 mM EDTA, 1 mM phenylmethylsulfonyl fluoride, 1 mM sodium orthovanadate, 10 mM NaF (all from Sigma-Aldrich), and the “Complete protease inhibitor mixture” (Roche Diagnostics, Monza, Italy) [53]. Nuclei were removed by centrifugation and after measuring total proteins concentration with Bradford assay (Bio-Rad Laboratories), proteins were separated by SDS-PAGE, blotted onto PVDF membrane (Bio-Rad Laboratories), and probed with primary antibodies [54].

The Purity™ anti-mouse/anti-rabbit detection system was used (Vilber, Eberhardzell Germany) and chemiluminescent detection (ChemiDoc™ Imaging System, BioRad Laboratories) was performed to visualize and quantify protein bands.

### Evaluation of Q54 and Q48 binding to CLIC1 protein

Microscale thermophoresis (MST) was used to assess Q48 and Q54 interaction with CLIC1 protein [55]. MST measurements were performed using Monolith NT.115p instrument (NanoTemper Technologies, Munich, Germany). Assays were conducted at 10–20% (BLUE/RED dye) LED excitation power and MST power of 40%. Premium capillaries from NanoTemper Technologies were used. Measurements were carried out at 25 °C in the following buffer: 10 mM HEPES (pH 8.00), 150 mM NaCl, 0.05% Tween20. Recombinant CLIC1 protein was labeled with the Monolith labeling kit RED-NHS (ammine dye NT-647-NHS) and with the Monolith labelling kit BLUE-NHS (ammine dye NT-495-NHS) according to manufacturer instructions (NanoTemper Technologies). MST detects the change in fluorescence of a labeled target along a temperature gradient induced by the activation of an IR laser, upon addition of a ligand [55]. Change in MST signal is expressed as the variation in the normalized fluorescence (Fnorm), defined as Fnorm = F1/F0, where F1 is the fluorescence after a given MST-laser on time and F0 the fluorescence prior to IR laser activation. ΔFnorm is the baseline-corrected normalized fluorescence, frequently expressed in parts per thousand [‰]. The affinity parameters Kd were determined by simultaneously performing the experiment on 16 capillaries, each containing a constant concentration of the labelled target (CLIC1) and increasing concentrations of unlabeled ligand (Q48 or Q54). The recorded gradual change in MST was then plotted as ΔFnorm against the ligand concentration to yield dose-response curves. Labelled CLIC1 concentrations used were 10 nM or 100 nM for RED or BLUE labelling, respectively. The highest concentrations tested for compound Q48 was 5 mM (1% of DMSO final) and 15 mM for Q54. Obtained data were fitted with sigmoidal models using the GraphPad Prism 5.0 software.

### Preparation of three-dimensional (3D) GSC cultures and proliferation assay

3D GSC organoids were obtained as previously described [56]. Briefly, 5000 cells were re-suspended in ice-cold Matrigel™, seeded on parafilm molds to obtain 20 μL droplets and incubated at 37 °C for 30 min. Droplets were gently removed from molds and transferred to 6-well plates in complete medium and incubated in 5% CO_2_ at 37 °C. Organoid morphology was monitored until dense aggregates are formed (about 7–10 days). Organoids were fixed in PFA for 1.5 h, at RT, washed with PBS and dehydrated overnight at 4 °C with 30% sucrose solution. To assess whole organoid structure, samples were permeabilized with 0.1% Triton X-100 for 20 min and blocked in 10% normal goat serum for 2 h, at RT, followed by overnight primary antibody incubation. Then, organoids were washed 4 times in PBS for 1.5 h each and incubated overnight with secondary antibodies AlexaFluor-568 and -488 (ThermoFisher Scientific). After further 4 washes in PBS 1x for 1.5 h each, fluorescent images were captured with Stellaris 8 TAU STED confocal microscope (Leica) and 3D images were generated using ImageJ software. Alternatively, organoids were frozen in OCT compound (Sigma-Aldrich), sectioned at 20 μM, and placed on microscope slides previously coated with gelatin (0.1%, Sigma-Aldrich) and chromium(III) potassium sulfate dodecahydrate (0.01%, Sigma-Aldrich). Slices were probed overnight with primary antibodies and nuclei were stained with DAPI following a standard immunofluorescence protocol, and then imaged with DM 2500 microscope equipped with DFC 350 FX digital camera (Leica).

Cell proliferation within treated organoids was assessed after 7 days in the absence or presence of 10 mM metformin, and 100 μM Q46, Q48, and Q54. Proliferating cells were detected using the Edu DetectPro Imaging kit 488 (Base Click GmbH, Neuried, Germany) [57], following the manufacturer’s protocol. Fluorescence was detected with Zoe™ Cell Imager (Bio-Rad Laboratories) and fluorescence intensity was measured on captured images using ImageJ software.

### RNA purification and quantitative real-time PCR

To extract RNA, organoids were treated with Cell Recovery solution (Corning, ThermoFisher Scientific) following the manufacturer’s protocol to allow Matrigel™ depolymerization. Total RNA was isolated from recovered cells using the PureLink RNA Mini Kit (Invitrogen). iScript™ Reverse Transcription Supermix was used for cDNA synthesis and cDNA products were analyzed using the SsoFast EvaGreen Supermix on a CFX96 Touch Real-Time PCR Detection System (all from Bio-Rad Laboratories).

Ribosomal protein large P0 (RPLP0) and 28S housekeeping genes, were used to normalize levels of target genes in each sample.

*Primers (human sequences):*

- CD44: forward = 5′-TGAATATAACCTGCCGCTTTG-3′; reverse = 5′-GCTTTCTCCATCTGGGCCAT-3′
- MAP2: forward = 5′-GAAAGACCAAGAGCCTACCACAG-3′; reverse = 5′-TCGGTCATGGCTTTCTCCAG-3′
- RPLP0: forward = 5′-TGTGGGCTCCAAGCAGATGCA-3′; reverse = 5′-GCAGCAGTTTCTCCAGAGCTGGG-3′
- 28S rRNA: forward = 5′-GTAACCCGTTGAACCCCATT-3′; reverse = 5′-CCATCCAATCGGTAGTAGCG-3′

### Zebrafish embryo husbandry, GSC grafting, drug treatment, and in vivo toxicity tests

#### Fish husbandry and embryo preparation

Zebrafish (*Danio rerio*) adults were raised and maintained following standard methods, according to National (Italian D.lgs 26/2014) and European (2010/63/EU and 86/609/EEC) animal welfare laws. A breeding stock of mature zebrafish was used for egg production: males and females (ratio 2:1) were kept in a tank under the following conditions: 26 °C ± 1 °C, 14 h/10 h light/dark cycle [58]. Spawning and fertilization took place within 30 min after induction by light. At 4–5 h post-fertilization (hpf), embryos were collected and rinsed with the culture medium to remove residues on the egg surface. Healthy embryos were selected for subsequent experiments. Within the timing in which embryos were analyzed, 120 hpf, experiments are not considered as animal experimentation according to Italian rules (Italian D.lgs 26/2014).

#### Patient-derived GSC orthotopic xenograft

Twenty-four hpf, wild type zebrafish embryos AB were soaked in embryo medium with 0.2 mM 1-phenyl 2-thiourea and incubated for further 24 h at 28.5 °C. At 48 hpf, embryos were dechorionated and anesthetized with 0.0003% tricaine prior to injection. Anesthetized embryos were positioned on a wet agarose 1% pad. The hindbrain of each embryo was injected with approximately 150–200 cells (ZsGreen-positive cells) using an Eppendorf FemtoJet microinjector, under the observation by stereoscope (MZ APO, Leica). After transplantation, embryos were incubated for 4 h at 32 °C and checked for the presence of fluorescent cells in the correct site. Then embryos were incubated at 32 °C in fresh medium for the following days. For the treatments, screened embryos were transferred in a 48-well plate with 1 mM compound concentration prior to the incubation at 32 °C. Five days post-fertilization (dpf, 3 days after injection), images of the tumors were captured and the relative integrated density, obtained by the product of the fluorescence intensity and the area of the tumor mass, was calculated as the ratio between the final and the initial tumor integrated density using ImageJ software.

#### In vivo toxicity

Test compounds were prepared immediately prior to use by diluting the stocks with the culture medium. Embryos were transferred to 6-well plates (20 embryos/well) in 3 ml of medium containing different concentrations of compounds or vehicle for control groups. Plates containing experimental embryos were placed in the laboratory with controlled light and temperature conditions.

The potential in vivo toxicity of the novel biguanide derivatives was assessed evaluating the effects on zebrafish embryo survival in comparison with those of metformin [59, 60]. Embryos were incubated with increasing drug concentrations up to 5-dpf, and Kaplan-Meier curves were generated to evaluate survival. Throughout the whole exposure period to compounds, the status of the zebrafish embryos was examined under a stereomicroscope: mortality, morphology of the body, heartbeat, movement, were recorded every 24 h. All dead embryos were readily removed to avoid well contamination. The endpoints used to assess toxicity included embryo survival/mortality confirmed visually by absence of heartbeat. Sub-lethal toxicity was qualitatively assessed by lack of startle response to a tactile stimulus (gentle touch on the dorsal surface with a fine pipette tip) and the presence of gross morphological abnormalities.

### In ovo toxicity

All activities related to the in ovo experiments, including the preparation of the final concentration of compounds, the incubation of eggs, the administration of test compounds, and the toxicity test, were carried out by the Inovotion lab team (Inovotion, La Tronche, France). All activities related to the in ovo experiments were conducted as previously described [61]. Briefly, Fertilized White Leghorn eggs (Société Française de Production Agricole, St.-Brieuc, France), were incubated at 38 °C with 60% relative humidity for 10 days. At stage E10, the chorio-allantoic membrane (CAM) was dropped by drilling a small hole through the eggshell into the air sac and a 1 cm^2^ window was cut in the eggshell above the CAM. Metformin and Q54 were incubated from day 9 to 19 of embryo development, being administered every 2 days. At the end of the treatment the percentage of alive/dead embryos was recorded ad analyzed as percentage of untreated eggs. Experiments were performed using an average of 20 eggs for each experimental group. According to French legislation, no ethical approval is needed for scientific experiments using oviparous embryos (decree no. 2013–118, February 1, 2013; art. R − 214 − 88).

### Dihydrofolate reductase (DHFR) assay

DHFR activity was performed using the Abcam colorimetric kit (ab239705) following manufacturer’s instructions. Spectrophotometric reading was performed at 30 °C, each 5 s, for 7 min, using a ClarioStar Plus microplate reader (BMG LabTech).

### Statistical analysis

All experiments, repeated at least three times and performed in triplicate or quadruplicate, were expressed as mean ± S.D./S.E.M. Statistical analyses and IC_50_ values, calculated using nonlinear regression curve fit analysis, were done using Prism 5.02 (GraphPad, San Diego CA, USA). Statistical significance between groups was assessed by t-test (unpaired, two-tailed) or one-way ANOVA followed by post-hoc Dunnett’s test, or Tukey multiple comparison test for zebrafish xenograft experiments. Selectivity Index was calculated as the ratio of IC_50_ of non-malignant astrocytes to IC_50_ of GSC cultures, as reported [62]. Survival proportions of zebrafish embryos were calculated using the Kaplan-Meier method and Log rank test for trend was used to compare survival curves. Statistical significance was established at *p* value ≤0.05.

## Results

### CLIC1 expression in human GBM samples

CLIC1 expression was initially analyzed in human GBM sections by immunohistochemistry. CLIC1 expression in GBM cells is diffuse and uniform within tumor tissue (Fig. S1A). An intense staining was also detected in subsets of cells, mainly localized close to the vessels (Fig. S1B, C), resembling infiltrating glial cells.

To measure CLIC1 expression in GSCs, transcript quantification by RNA-Seq was carried out in a panel of 14 cultures. Overall, average gene expression across GSCs showed high levels of CLIC1 mRNA, although reflecting the expected heterogeneity among distinct patient-derived cultures (Fig. S2A). RNA-Seq results were validated by Western blot, in selected samples to be used in further experiments (Fig. S2B). In this screening we identified only one GSC culture, derived from GBM39, which expresses very low CLIC1 mRNA content, although retaining malignant tumor features similarly to CLIC1-expressing GSC cultures (Table S1).

### Synthesis of biguanide compounds

**Fig. 1:**
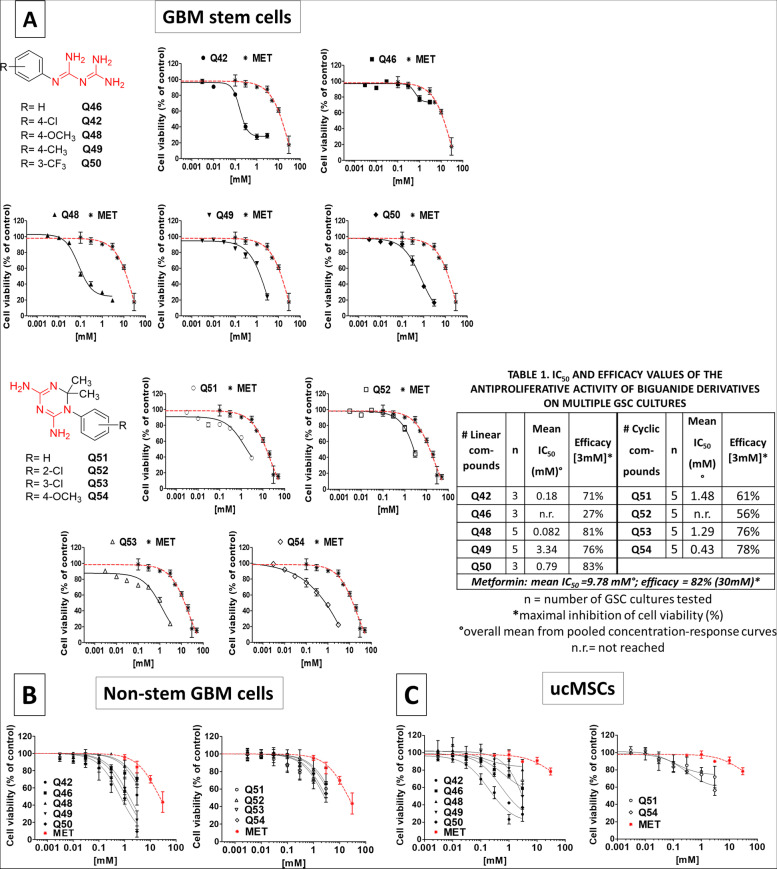
**A** Concentration-response curves of novel biguanide derivatives on GSC viability, in comparison with metformin activity. UPPER LINES (from the left): Chemical structures of linear biguanides (structurally related to metformin) highlighting the biguanide moiety (in red); antiproliferative activity of Q42, Q46, Q48, Q49, and Q50 in comparison with metformin activity. LOWER LINES (from the left): Chemical structures of cyclic biguanides (structurally related to cycloguanil) highlighting the biguanide moiety (in red); antiproliferative activity of Q51, Q52, Q53, and Q54 in comparison with metformin activity. Cell viability was evaluated by MTT assay after 48 h of treatment. Data are reported as average of replica experiments in multiple GSC cultures (mean ± S.E.M. of at least three independent experiments for each culture). Table 1 within the figure reports the number of cultures analyzed for each compound and the calculated potency and efficacy. Compound effect in individual cultures is reported in Fig. S3, and point-by point statistical analysis is reported in Table S3. **B** Concentration-response curves of novel biguanide derivatives on differentiated (non-GSCs) glioblastoma cell viability, in comparison with metformin activity. Left graph: antiproliferative activity of Q42, Q46, Q48, Q49, and Q50 in comparison with metformin activity (in red). Right graph: antiproliferative activity of Q51, Q52, Q53, and Q54 in comparison with metformin activity (in red). Cell viability was evaluated by MTT assay after 48 h of treatment. Data are reported as average of replica experiments in multiple differentiated glioblastoma cell cultures (mean ± S.E.M. of at least three independent experiments for each culture), obtained by the same GSC cultures analyzed in A, by shifting culture conditions in FBS containing medium. Compound effect in individual cultures is reported in in Fig. S4, and point-by point statistical analysis is reported in Table S4. **C** Concentration-response curves of novel biguanide derivatives on umbilical cord mesenchymal stem cell (ucMSC) viability, in comparison with metformin activity. Left graph: antiproliferative activity of Q42, Q46, Q48, Q49, and Q50 in comparison with metformin activity (in red). Right graph: antiproliferative activity of Q51, Q52, Q53, and Q54 in comparison with metformin activity (in red). Cell viability was evaluated by MTT assay after 48 h of treatment. Data are reported as average of replica experiments in independently isolated ucMSC cultures (mean ± S.E.M. of at least three independent experiments for each culture). Compound effect in individual cultures is reported in in Fig. S5, and point-by point statistical analysis is reported in Table S5

The high percentage of GSC cultures expressing CLIC1, and the known ability of biguanides to act as CLIC1 blockers, prompted us to screen the efficacy of novel biguanide derivatives. In particular, metformin and cycloguanil core structures were selected as valuable chemotypes, to be optimized for the development of novel anticancer agents [38]. To adequately tune CLIC1 inhibition, we synthesize two series of compounds: aryl-biguanides (named Q42, Q46, Q48, Q49, and Q50) and cycloguanil-like derivatives (Q51, Q52, Q53, and Q54) introducing substitutions on aromatic rings characterized by opposite electronic properties and polarity. Molecular structures of novel linear and cyclic biguanides are depicted in Fig. 1A.

The likely pharmacokinetic behavior of the ionizable form of these compounds has been predicted calculating pKa and lipophilicity LogD descriptors (Table S3). A small improvement as compared to metformin, as far as the linear compounds was observed, while cycloguanil and the novel cyclic molecules showed similar values, indicating that, as occurs for metformin, simple diffusion through membranes is rather inefficient, and BBB crossing mainly occurs via carrier-mediated active transport.

### Antitumor activity of the novel biguanide-based compounds on GSCs

#### Antiproliferative activity of biguanide compounds

Antiproliferative efficacy of the novel biguanide-based compounds, in comparison with metformin used as reference drug, was tested on CLIC1-expressing GSC cultures, according to data reported in Fig. S2. To overcome potential biases due to GBM heterogeneity, molecules were tested on five independent GSC cultures (GBM3, 5, 19, 23, and 37). Cell viability was assessed by MTT assay after compound exposure for 48 h, in concentration-response experiments. Data are reported as overall mean effects on all cultures in Fig. 1 (for sake of clarity, statistical significance for each point is reported in Table S4) and as individual tumor response in Fig. S3. IC_50_s for both linear and cyclic biguanides are listed in Table 1 within Fig. 1A.

Metformin inhibited cell proliferation confirming high efficacy and low potency (max. Inhibition − 82% and average IC_50_ 9.78 mM, Table 1) in agreement with previous reports [38]. Concentration-response curves of the novel biguanide derivatives significantly differed from metformin as far as potency, since four compounds (Q42, Q48, Q50 and Q54) showed mean IC_50_ values within the μM range, three (Q49, Q51 and Q53) in the low mM range, and only two (Q46 and Q52) did not reach an adequate inhibitory activity to allow the calculation. In particular, the best performing molecules, i.e. exhibiting the highest potency, were the linear compound Q48 (mean IC_50_ = 0.082 mM) and the cyclized analogue Q54 (mean IC_50_ = 0.43 mM).

Regarding the efficacy of each compound, assessed comparing the maximal antiproliferative effect observed at 3 mM, all active biguanides (Q42, Q48, Q49, Q50, Q53 and Q54) caused a highly significant inhibition of GSC viability, showing efficacy ranging between − 71 and − 83%. Q46 (− 27%), Q51 (− 61%) and Q52 (− 56%) were the least effective compounds (Table 1). Metformin also showed high efficacy (− 82%) but required 30 mM concentration to yield the maximal effect, which is 10-fold higher than that of the novel derivatives. Importantly, all tested GSC cultures showed similar sensitivity to all biguanides (Fig. S3), and the expected slight variability was not statistically significant.

Metformin antiproliferative activity in GSCs is mostly dependent on CLIC1 inhibition, whilst loses efficacy in cells in which CLIC1 activity is not required for cell-cycle progression, such as non-stem GBM cells (differentiated cells obtained from GSC cultures shifted in FBS-containing medium) and primary cultures of ucMSCs [6, 27, 38]. Thus, we verified the target specificity of the novel biguanides derivatives measuring their antiproliferative activity (0.03-3 mM for 48 h) on differentiated GBM cells and ucMSCs by MTT assay.

In differentiated cells, minor antiproliferative efficacy was obtained using Q46, Q48, Q51, Q52, Q53 and Q54, when used within the IC_50_ range, which induced a significant inhibition of proliferation in GSCs isolated from the same GBM (Fig. 1B, Fig. S4 for data on individual cultures, statistical analysis is reported in Table S5). Conversely, Q42, Q49 and Q50 markedly dampened non-stem GBM cell proliferation with similar potency and efficacy observed in GSCs. Similarly, in four independently isolated ucMSC cultures, Q42, Q49 and Q50 impaired cell viability in a concentration-dependent manner with IC_50_ values of 0.19, 8.02 and 0.52 mM, respectively, while Q46, Q48, Q51, Q52, Q53 and Q54 exhibited a slight inhibitory effect without reaching at least 50% inhibition, as also observed using metformin (− 40% at 50 mM) (Fig. 1C, data from individual cultures is reported in Fig. S5, and statistical analysis in Table S6).

These results clearly indicate that Q42, Q49 and Q50 effectiveness was not (or not only) mediated by CLIC1 inhibition, and suggest that other targets, relevant for non-tumor stem cell survival are involved, which may possibly cause systemic toxicity, when used in patients.

On these bases, we excluded the compounds with high off-target activity (Q42, Q49 and Q50) or displaying modest efficacy (Q46, Q51 and Q52) and selected, for more in-depth investigations, the two compounds (one linear, Q48, and one cyclic, Q54) ensuring the best performance in terms of higher potency, efficacy, and lower toxicity towards ucMSCs; moreover, metformin and Q46 were also included in the following experiments as positive and negative controls, respectively.

#### In vivo toxicity of the novel biguanides derivatives

One of the best metformin features, pushing its repositioning as anticancer drug, is the combination of antiproliferative activity and low systemic toxicity, as demonstrated by its widespread use as hypoglycemic agent [63]. Thus before detailing the antitumor activity of the novel biguanide derivatives, we checked their in vivo toxicological profile in comparison to metformin, using zebrafish embryos. Zebrafish embryo is an efficient experimental model ensuring convenient drug delivery and retaining the biological complexity of vertebrate systems [64]. Embryos were treated for 5 days with growing concentrations comprising IC_50_ values of each drug: metformin (1-10 mM), Q48 (0.03-1 mM) and Q54 (0.1-10 mM). Lethal and sub-lethal endpoints were microscopically inspected and recorded. Treated embryos did not show visible developmental defects or phenotypic changes within 5-day exposure to these compounds. Kaplan-Meier’s plot revealed limited toxicity only for concentrations significantly higher than the respective antitumor IC_50_s (Fig. S6). In detail, exposure to the highest concentration of Q48 (1 mM) showed 62% survival after 5 days, with survival curves superimposable to control groups (log rank test for trend *p* = 0.62), demonstrating a non-significant impact on embryo survival. Similarly, Q54 had no toxic effects at concentrations up to 10 mM, with survival curves non-different from controls (log rank test for trend *p* = 0.64). Importantly, toxicity induced by Q48 was comparable to that induced by metformin, which decreased the survival of embryos (52%), while the effects of Q54 were even lower, but they did not differ from one another (log rank test for trend *p* = 0.38).

Similar results were obtained using chick embryos, which did not display signs of toxicity after 10-day incubation with concentrations of metformin and Q54 up to 3 mM (Fig. S7). Overall, these data predict the potential safety of these compounds even when used at high concentrations, although a safer profile for Q54 was identified.

#### Effect of novel biguanide-based compounds on proliferation rate and CLIC1-mediated ion current in GSCs

**Fig. 2:**
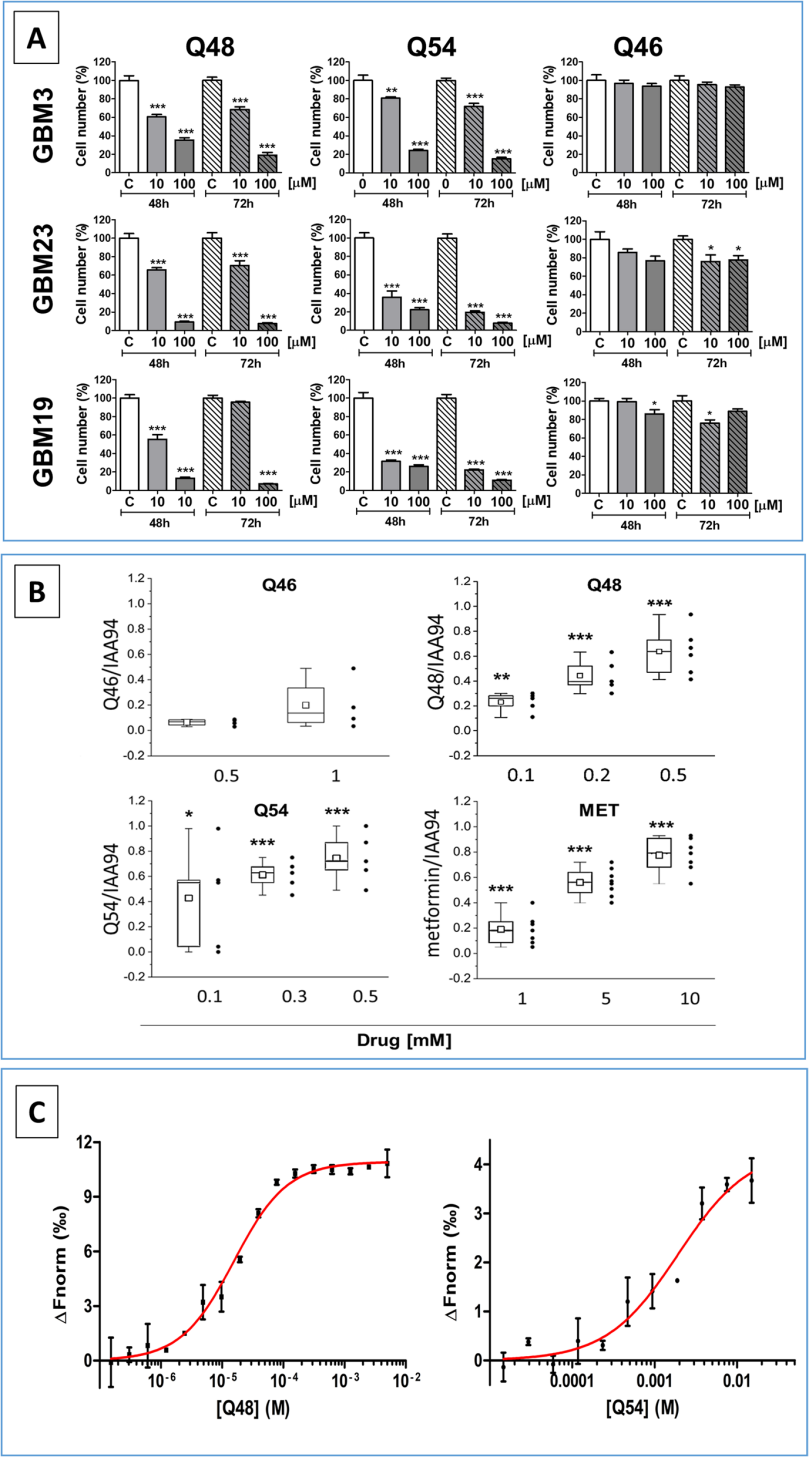
**A** Antiproliferative activity of Q48, Q54, and Q46 biguanide derivatives in GSCs, evaluated by live cell counting. Cell proliferation of GBM3, 19 and 23, treated with Q48, Q54 and Q46 at 10 and 100 μM concentrations was evaluated counting live cells by Trypan-blue exclusion, 48-72 h post-treatment. Data represent the mean ± S.E.M. of 3 independent experiments performed in quadruplicate. Direct cell counting confirmed the high efficacy and potency of Q48 and Q54. Q46, used as negative control, was ineffective. *p < 0.05, **p < 0.01, ***p < 0.001 vs. respective control (CTR). **B** Effect of novel biguanide derivatives on CLIC1-mediated ion current in GSCs. CLIC1-mediated ion current was evaluated by whole cell patch clamp electrophysiology measurement in voltage-clamp configuration in GBM3. Cell were sequentially perfused with vehicle (controls), Q46 (0.5–1 mM), Q48 (0.1–0.5 mM), Q54 (0.1–0.5 mM) and metformin (1–10 mM), followed by IAA94 (100 μM) to identify CLIC1 residual activity, in current time-course experiments. Box chart plots report the mean current inhibition as ratio of the biguanide and IAA94 sensitive currents. IAA94 treatment represents the residual CLIC1 activity. Grey dots, next to box charts, indicate the number of cells used for the statistics (range 4–8) for each drug concentration. *p < 0.05, **p < 0.01, ***p < 0.001 vs. respective control cells. **C** Microscale Thermophoresis (MST) analysis of CLIC1 binding by novel biguanide compounds. MST analysis of Q48 (left) and Q54 (right) binding to CLIC1. Titration curve of RED-NHS-labelled CLIC1 (10 nM) with increasing concentrations of novel compounds. MST data are the average of three replicates. The sigmoidal fitting curve, obtained using GraphPad Prism 5.0 software, is shown in red

Antiproliferative efficacy of Q48, Q54, and Q46 (10-100 μM) was directly measured on GSC growth rate, comparing viable cell number after 48-72 h treatment of GBM3, GBM23 and GBM19. By means of Trypan blue exclusion assay (Fig. 2A), we observed that both concentrations of Q48 and Q54 significantly decreased viable cell number, in all the GSC cultures. Maximal efficacy was reached using 100 μM at both 48 and 72 h, with a percentage of viable cells reduced by 75–93% with Q48 and by 76–92% with Q54. Q46 confirmed its weak activity in all the cultures. Specificity of the antiproliferative activity of Q54 and Q48 against GSCs was further confirmed demonstrating the lack of effects on cultured normal astrocytes up to the concentration of 3 mM. Q46 was ineffective also toward astrocytes, while metformin reduced viability (− 50%), but only at very high concentrations (30 mM) (Fig. S8A). Comparison of the IC_50_ obtained in normal astrocytes and GSCs allowed us to define the selectivity index [62] of each compound, which is represented by the ratio of the toxic concentration of a sample against its effective bioactive concentration. Q48, Q54 and metformin displayed a selectivity index > 10 (246, 52, and 11, respectively, Fig. S8B), indicating a specific antiproliferative activity against tumor cells as compared to normal cells.

Then we checked the direct effects of these biguanide derivatives on CLIC1 activity, evaluating CLIC1-mediated ion current in perforated patch clamp experiments. GBM3 GSCs were sequentially perfused with vehicle (controls) or different concentrations of Q46, Q48, Q54 and metformin, followed by the CLIC1 inhibitor IAA94, in current time-course experiments. Box plots in Fig. 2B report the percentage of current inhibition as ratio of the inhibition induced by each compound and by IAA94, to identify biguanide-insensitive CLIC1 current, since 100 μM IAA94 completely blocks CLIC1 current [65]. These experiments (Fig. 2B) show that Q48 and Q54 concentrations corresponding to IC_50_ and above, reduced CLIC1 activity in a concentration-dependent manner, although only Q54 experienced an efficacy comparable to metformin (about − 80% of CLIC1 current) for concentrations corresponding to the antiproliferative IC_50_ (Table 1 in Fig. 1). Moreover, Q54 displayed higher potency than metformin, achieving maximal inhibition at 0.5 mM vs. 10 mM of metformin. Conversely, Q48 inhibited by 50% CLIC1 activity at the concentration of 0.2 mM, which is more than double of the antiproliferative IC_50_ (Table 1), and a complete CLIC1 inhibition occurred only at the concentration of 0.5 mM. Thus, differently from Q54, whose antiproliferative activity was strictly correlated to a complete CLIC1 inhibition, Q48 exerted a maximal inhibition of GSC proliferation at concentrations in which CLIC1 current is not completely blocked, suggesting that other molecular targets may contribute to its antiproliferative effect. Finally, as expected from cell viability data, Q46 did not affect CLIC1 activity at concentrations up to 1 mM.

The direct interaction of Q48 and Q54 with recombinant CLIC1 protein was tested through MST experiments by labelling CLIC1 with fluorescent dyes covalently bound to the primary amines (lysine residues) of the protein. Thermophoretic-induced fluorescence change of the labelled-target was detected upon addition of increasing concentrations of the two compounds. All data were collected right after sample preparation. A dose response curve was built for each compound, providing affinity parameters of Kd = 15.6 ± 1.9 μM and 1.9 ± 0.5 mM, for Q48 and Q54, respectively (Fig. 2C), in line with the IC_50_ above reported (Table 1). Data reported in Fig. 2C for CLIC1 labelled with a red fluorescent dye were confirmed by the same experiments performed by labelling the protein with a blue fluorescent dye (data not shown).

The specificity of CLIC1 as target for biguanide antiproliferative activity was further supported in GBM19 GSCs in which CLIC1 expression was down-regulated by RNA interference (siCLIC1 cells, Fig. S9A). Contrarily to what observed in siLuc control cells, Q48 and Q54 are unable to significantly reduce siCLIC1 cell proliferation (Fig. S9B). However, we have to point out that CLIC1 silencing almost abolished cell proliferation (135% vs. 258% of time 0, in siCLIC1 and siLuc cells, respectively, 48 h after plating), making difficult to evaluate a possible further reduction of the proliferation rate.

To assess the possible involvement of different intracellular pathways in biguanide effects in GSCs, in light with previous studies [66] we analyzed by WB the effects of Q48, Q54, and metformin on ERK1/2 activation induced by bFGF (40 ng/ml) treatment. However, we observed different effects in different GBM cultures (Fig. S10). In particular, Q48 and Q54 were able to inhibit ERK1/2 activation in GBM 3 and 19, and metformin only in GBM 3; conversely GBM 23 was not responsive to all the tested compounds. Thus we did not identify any relationship between biguanides’ effects as far as inhibition of ERK1/2 activity and the antiproliferative effects, further supporting the specificity of CLIC1 activity inhibition in the antitumor activity of the tested compounds.

#### Effect of the novel biguanides on GSC sphere formation

Q48 and Q54 were then tested by sphere formation assay, as indirect index of GSC stemness and self-renewal capacity.

**Fig. 3:**
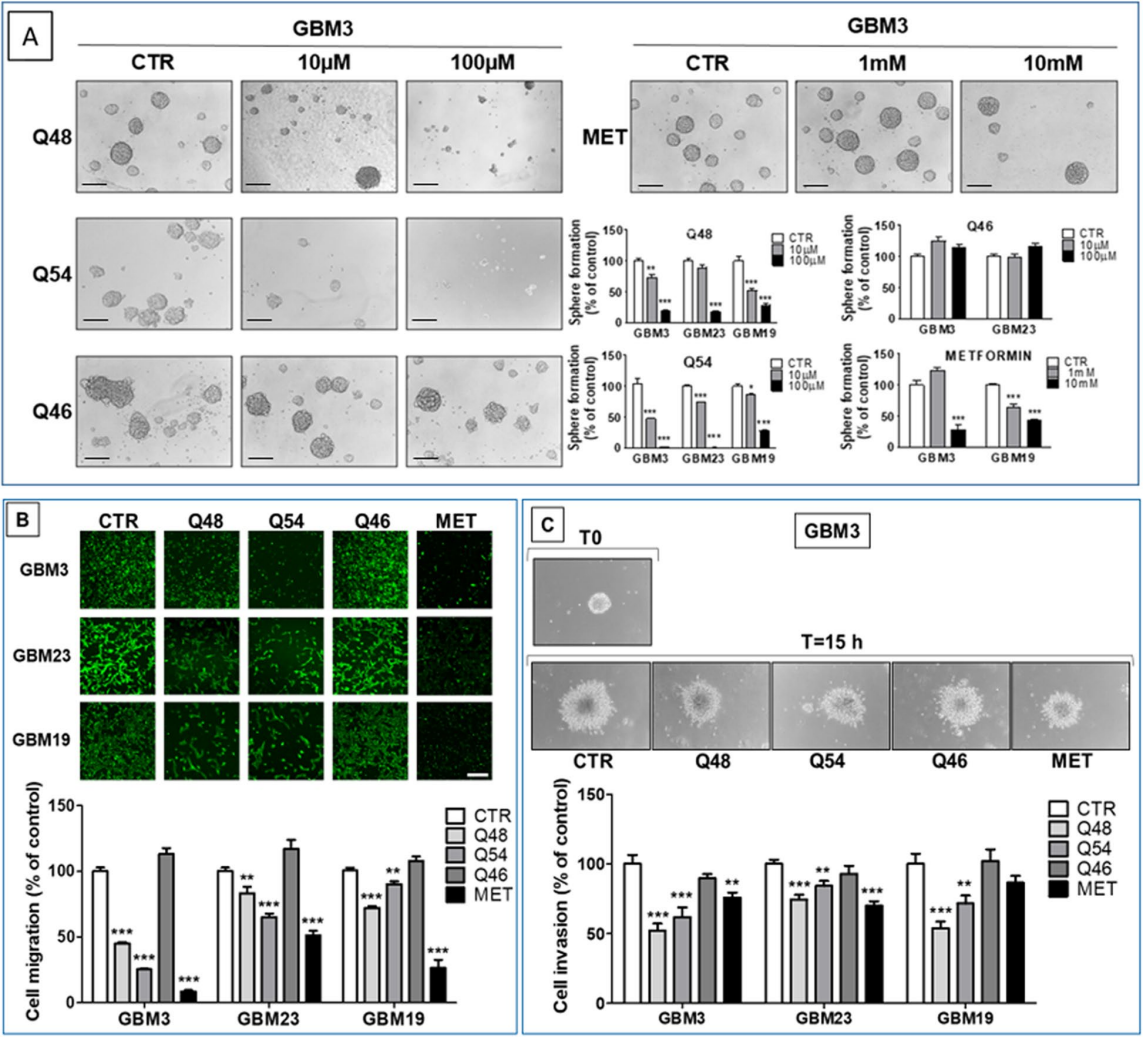
**A** Effect of novel biguanide derivatives on GSC sphere formation ability. Sphere-formation assay was performed on GBM3 GSCs, treated with Q48, Q54, and Q46 (10–100 μM) or metformin (1-10 mM) for 7 days, and analyzed by microscopic assessment and imaging. Spheres were counted by two independent investigators and the percentage of sphere-forming cells determined for each treatment group (*n* = 3). Representative phase contrast microscopic images of serum-free suspension depicting number and size of spheres in control (CTR) and Q48, Q54, and Q46-treated (10 and 100 μM) GBM3 cells. Compounds efficacy is compared to metformin (MET) activity, used at 1 and 10 mM. Bar = 150 μm. Bar graphs report sphere number generated after compound treatments, expressed as the mean percentage ± S.D. of respective CTR. *p < 0.05, **p < 0.01, ***p < 0.001 vs. CTR. **B** Effect of novel biguanide derivatives on GSC migration. Fluorescently labeled cells from GBM3, 19, and 23 were treated with Q46, Q48 and Q54 (100 μM), metformin (10 mM), or vehicle (CTR). GSCs were plated in fluorescence-blocking membrane transwells, using 10% FBS-containing medium as chemoattractant in the lower wells. Migrated cells reaching the bottom surface of the membrane were photographed by confocal microscopy and quantified by ImageJ software. Left panels: Representative confocal microscopy images of migrated cells on the surface of the permeable support. Bar = 100 μm. Right panels: Quantification of migrated cells using ImageJ. Data represent the mean ± S.D. (*n* = 3). **p < 0.01, ***p < 0.001 vs. CTR. **C** Effect of novel biguanide derivatives on GSC Matrigel invasion. GBM3, 23 and 19 newly formed spheres were embedded in Matrigel and treated with Q48 (100 μM), Q54 (100 μM), Q46 (100 μM) and metformin (10 mM). The invasion rate was evaluated after 15 h, using the ImageJ software, measuring at least 2 diameters for each sphere. The differences between the average diameter of T15 and T0 of each condition were calculated and compared to control. Upper panels: Representative GBM3 images of individual sphere embedded in Matrigel at T0 and after 15 h of treatment (magnification: 10x). Lower panels: Bar graphs represent the mean ± S.E.M (*n* = 25), expressed as percentage of respective controls (CTR) of cell invasion. ∗*p* < 0.05, ∗∗*p* < 0.01, ∗∗∗*p* < 0.001 vs. respective CTR

Exposure of GBM3, GBM23 and GBM19 cultures to Q48 and Q54 (10 and 100 μM) for 7 days nearly abolished sphere formation, reducing both number and size (Fig. 3A), while Q46 was not effective. Q54, which displayed the highest CLIC1 inhibition, shows a statistically significant inhibition of sphere formation in all GSC cultures tested already at 10 μM, and a complete abolishment at 100 μM. These data demonstrate that Q54 potency in reducing self-renewal is higher than its activity on GSC proliferation, which occurred with an IC_50_ of 430 μM. Ten μM Q48 showed lower efficacy than Q54 and did not accomplish statistical significance in GBM23 GSCs; however, using Q48 at the antiproliferative IC_50_ (100 μM) a highly significant inhibition of spherogenesis was observed (up to 83% in GBM23, 81% in GBM3 and 75% in GBM19). In comparison, metformin impaired sphere-forming GBM3 and GBM19 (− 73% and − 57%, respectively) when used at 10 mM, reflecting its antiproliferative IC_50_ (Fig. 3A).

#### Effect of novel biguanide derivatives on GSC migration and invasion

GSC infiltration into surrounding brain is a GBM hallmark and represents a major cause of relapse. Therefore, we investigated the impact of Q48 and Q54 on GSC migration, in a transwell assay, tracking fluorescently labelled cell movement toward FBS-containing medium, used as chemoattractant. In control conditions, GBM3, GBM23 and GBM19 GSCs showed high motility toward FBS-containing medium. In the presence of Q48 or Q54 (100 μM), migration slowed-down in all GSC cultures, with a maximal inhibition observed in GBM3 (− 66% for Q48 and − 75% for Q54, Fig. 3B). Metformin also inhibited GSC migration showing higher efficacy than the novel compounds, although, as expected, required a higher concentration (10 mM) to be effective. Again, Q46 was ineffective in all the tested cultures.

More importantly, the new biguanide derivatives Q48 and Q54 (but not Q46), as well as metformin, were able to inhibit GSC invasion from spheroids into Matrigel™ (Fig. 3C), with similar effects observed in cultures derived from 3 GBMs (GBM3, GBM19, GBM23). Statistical significance for the anti-invasive activity was reached in all the GBM cells treated with both Q48 and Q54 (100 μM), although the former showed higher efficacy. Metformin (10 mM) significantly inhibited cell invasion in GBM3 and GBM 23, although in GBM19 the inhibitory effects did not reach the statistical significance. Q46, used as negative control, was completely ineffective in all the GSC cultures analyzed.

#### Biguanide derivatives affect proliferation of 3D GSC cultures

In vitro 3D GBM cultures (organoids) better mimic tumor tissue structure, cell-to-cell and cell-to matrix interactions occurring in vivo, and more accurately predict drug efficacy than 2D cultures [67]. Isolated GBM3 GSCs were grown in 3D embedded in Matrigel™ droplets [56], and organoid proliferation was assessed by Edu-green fluorescent thymidine analogue labeling, which detects cells undergoing DNA synthesis [57].

**Fig. 4:**
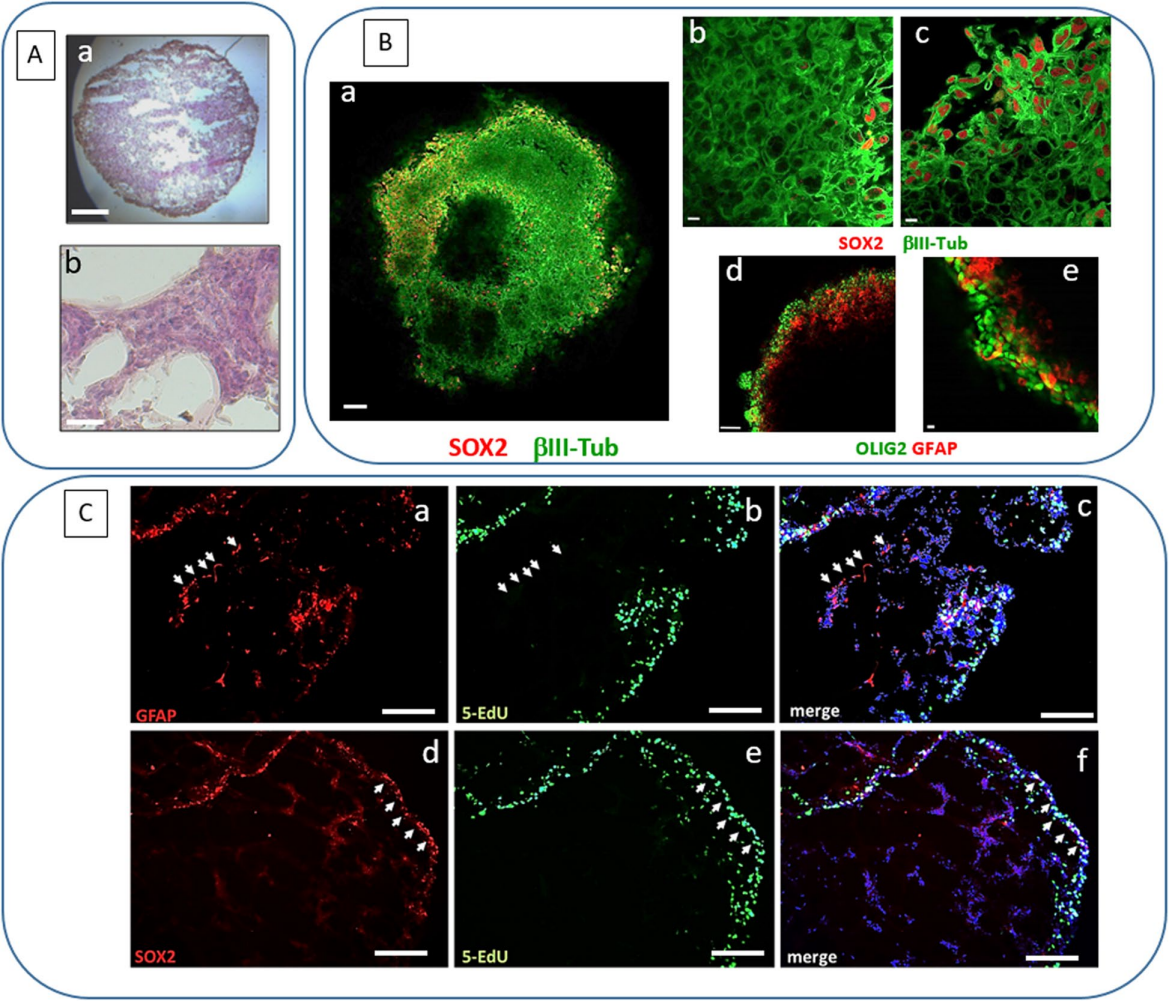
Characterization of GSC 3D organoids. **A** Hematoxylin & eosin staining of GSC organoid sections, after 3 weeks of cultures: cells are organized in a tissue-like layered structure. a: bar = 500 μm; b: bar = 50 μm. **B** Immunofluorescence images of the whole GSC organoid structure labelled with Sox2 (red) and β-III tubulin (green) (a: bar = 100 μm; b and c: bar = 10 μm), or Olig2 (green) and GFAP (red) (d: bar = 100 μm; e: bar = 10 μm) obtained by confocal microscopy. Cells expressing stem cell markers, Sox2 and Olig2, display a peripheral localization as compared to differentiated cells expressing β-III tubulin and GFAP. **C** Immunofluorescent labelling for GFAP (red, upper pictures) and Sox2 (red, lower pictures) performed on slices of GSC organoids after 3 weeks of cultures, incubated with 5-EdU (green), to mark actively proliferating cells. Nuclei are counterstained with DAPI (blue). White arrows highlight that most proliferating cells are Sox2-positive and localized at the periphery of the organoid; conversely, most GFAP-positive cells display an inner localization and are not co-labelled by 5-EdU. Bar = 200 μm

Initially, we characterized the phenotypical features of cells composing 3D cultures in comparison with 2D ones. GSC-derived organoids developed a tissue-like layered cellular organization Fig. 4A), with cells retaining stem-like marker expression (i.e. Sox2 and Olig2), similarly to the GSCs used to generate the organoids, mainly composing the marginal layers (Fig. 4B). Conversely, internal layers were constituted by differentiated GBM cells, expressing, β-III tubulin or GFAP (Fig. 4B) suggesting that the 3D structure favors the development of cellular heterogeneity typical of GBMs. mRNA analysis by RT-PCR confirmed that GSCs undergo differentiation within organoids, showing that CD44, a cell surface protein involved in cell-to-matrix and cell-to-cell interactions, which is barely expressed in 2D grown cells, became highly expressed in 3D cultures analyzed 15–30 days after embedding in Matrigel™ pearls (Fig. S11A). Similar results were observed for MAP2 expression (Fig. S11B). The acquired heterogeneity of the 3D cultures, resembling in vivo GBM phenotype, supports the notion of the high predictive validity of organoids for the screening of antitumor drugs [68].

Importantly, proliferating cells (Edu green-labeled cells) were mainly located in the external layers, showing a GSC-like phenotype, as for Sox2 expression, while differentiated internal cells, expressing GFAP, were mainly, non-proliferating (Fig. 4C).

**Fig. 5:**
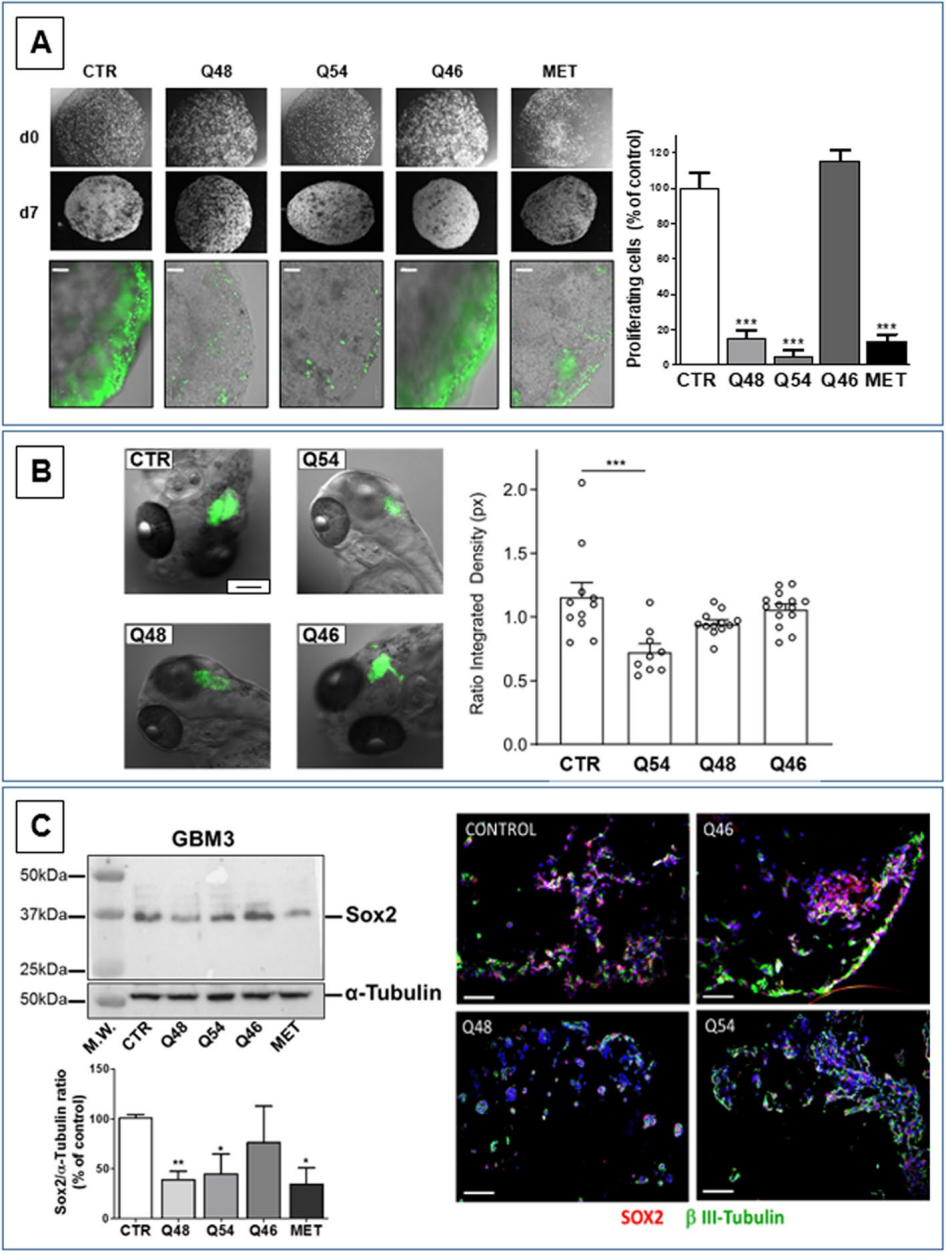
**A** Q48 and Q54 and metformin, display antiproliferative activity in the GSC organoids. Representative images of organoids at d0 and d7, prior to treatment (upper pictures). After further 7 days of treatment proliferating cells were labelled with 5-EdU (green in the lower pictures). Bar = 100 μm. The quantification of the compound-dependent changes in proliferation rate (reported as % of control values in the graph) was obtained using the ImageJ software. Q48 and Q54 (100 μM) and metformin (10 mM) caused a highly significant reduction of proliferating cells, while Q46 was not effective. ***p < 0.001 vs. CTR. **B** In vivo antitumor activity of novel biguanides in zebrafish xenotransplanted with GCSs. Left: Representative pictures showing the expansion of the tumor mass at 72 h post injection in zebrafish embryos’ brain injected with ZsGreen-positive cells treated with 1 mM Q54, Q48, and Q46 dissolved in embryos’ water. CTR = controls. Scale bar 100 μm. Right: Quantification of the integrated density of the tumor mass in control condition or in presence of the compounds. Every experimental point represents the expansion of the tumor mass measured in the single embryo’s brain. Data are reported as mean ± SEM; CTR *n* = 11, Q54 *n* = 9, Q48 *n* = 12, Q46 = 14; ***p = 0 .0002. **C** Biguanide treatment selectively reduce Sox2^+^ cell content. Left: Western blot depicting Sox2 levels in GBM3 GSCs cultures in control conditions (CTR) or after treatment with with Q48 (100 μM), Q54 and Q46 (300 μM), and metformin (10 mM) for 48 h α-tubulin analysis was used to normalize the results for protein content (upper panel). All the treatments, but Q46, significantly reduced Sox2 expression. Quantification by densitometric analysis normalized for α-tubulin expression, reported as the average of two independent analyses (lower panel). * = *p* < 0.05; ** = *p* < 0.01. Right: Representative images of the changes in cell composition within organoids after Q46, Q48, and Q54 treatment evaluated by immunofluorescence, labelling GSCs (Sox2-expressing, red) and differentiated cells (β-III tubulin-expressing, green). Nuclei are counterstained with DAPI (blue). Bar = 200 μm. Q48 and Q54 treatment significantly reduced the number of Sox2-expressing cells (purple for the co-localization of Sox2, red, and nuclei, blue) as compared to vehicle-treated control. Q46 is ineffective

Organoids were treated for 7 days with the novel biguanides, and assayed for proliferation activity in comparison with vehicle-treated 3D cultures (Fig. 5A). Q54 and Q48 (100 μM) significantly reduced the number of cells in active DNA synthesis by 95 and 85%, respectively, while metformin required 10 mM concentration to induce similar effects (− 83%). Q46 (1 mM) did not affect organoid proliferation. To compare the relative potency of novel biguanides on GSCs grown in 3D, we disaggregated treated and control spheroids obtained from GBM 3, 19, and 23, and calculated efficacy and IC_50_ values from dose-responses curves by MTT assay. Q48 and Q54 confirmed a higher potency than metformin, with the calculated IC_50_ values only slightly different from what observed in 2D. Q48 displayed a 5-fold reduction in IC_50_ (from 0.082 to 0.43 mM), while no significant differences occurred for Q54 (0.43 vs. 0.4 mM). However, as expected, drug efficacy (i.e. the average maximal inhibition occurring in the 3 GSC cultures at 3 mM of each compound) detected in 3D cultures was significantly reduced in comparison to the maximal antiproliferative effect detected in 2D (*p* < 0.01): − 39, − 54%, and − 55% vs. -81, − 78%, and − 82% (see Table 1), for Q48, Q54 and metformin, respectively.

#### Effect of Q48, Q54 and Q46 on in vivo GSC-dependent tumor growth using the zebrafish model

To test the in vivo efficacy of the novel compounds, we evaluated tumor growth after ZsGreen labeled GBM3 GSC xenotransplant in zebrafish hindbrain (Fig. 5B). Q48, Q54, and Q46 (1 mM), were added to culture water for 3 days and tumor growth evaluated measuring fluorescence intensity and area of the tumor mass using ImageJ software. Q54 caused a highly significant (*p* = 0.0002) reduction of tumor growth, confirming its in vitro antitumor efficacy, while Q46 was unable to affect GSC growth in vivo as it did in vitro. Conversely, Q48, which was highly effective in vitro, slightly reduced GBM3 tumor mass without reaching statistical significance.

#### Selectivity of novel biguanide antitumor effect toward GSCs in 2D and 3D tumor growth models

To further demonstrate the selectivity of biguanides for GSCs, we treated 2D and 3D cultures from GBM3 with Q48 (100 μM), Q54 (300 μM), and metformin (10 mM) for 48 h, and evaluated Sox2 expression by Western blotting (2D cultures, Fig. 5C left panels), and the presence of Sox2^+^ cells by IF (3D cultures, Fig. 5C right panels). Both novel compounds and metformin reduced Sox2 levels in 2D cultures, clearly indicating a depletion of stem-like cells after treatment. Similarly, Q48 and Q54 caused a reduction of Sox2-expressing cells in GBM organoids. Importantly, in both experimental models no changes in Sox^+^ cells were observed after Q46 treatment, confirming the specificity of the observed effects (Fig. 5C).

### Low CLIC1 expression negatively influences in vitro proliferation but not stemness and tumorigenic potential of GSCs

**Fig. 6:**
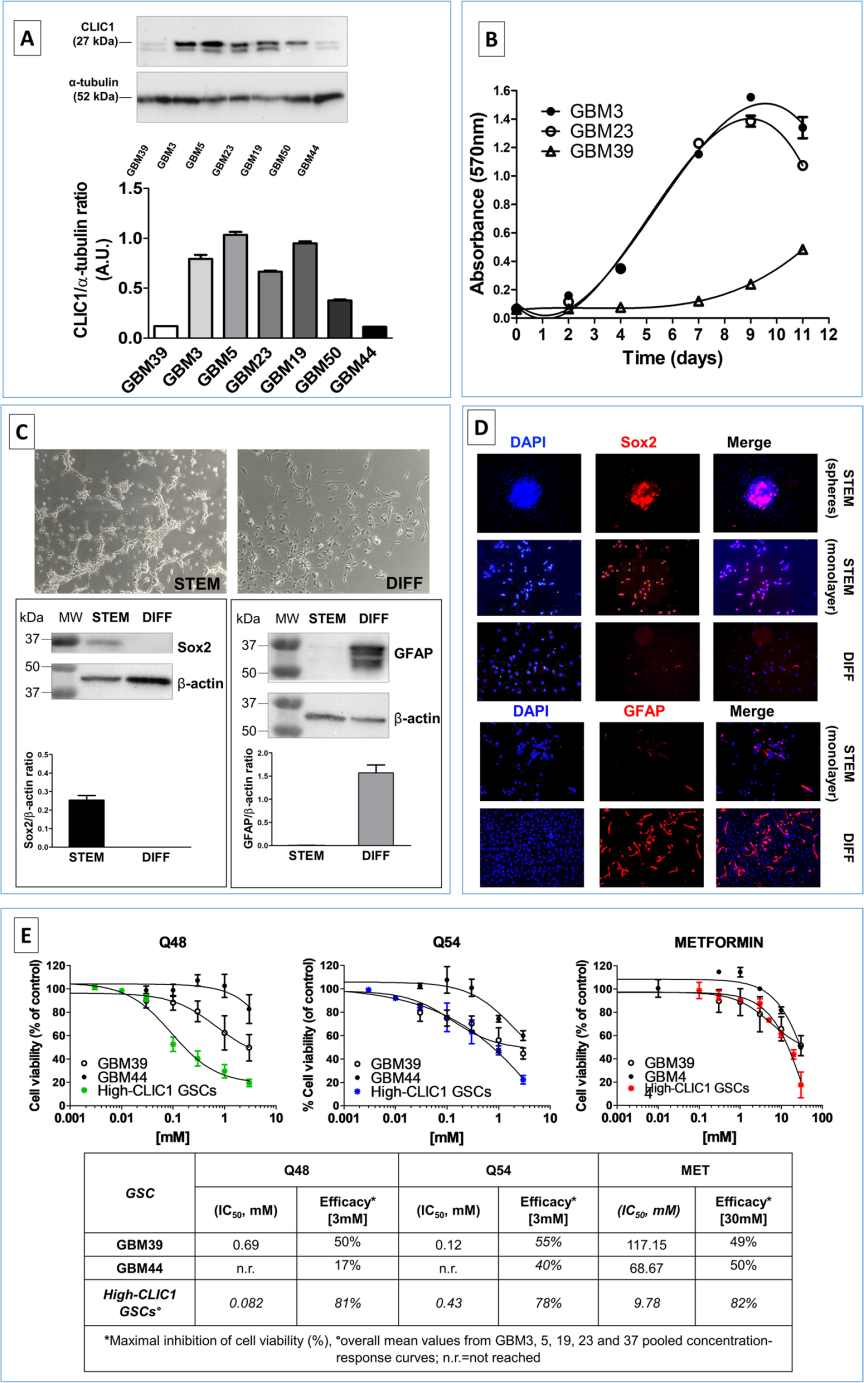
Characterization of GSC cultures expressing low levels of CLIC1 protein. **A** Selected GSC cultures expressing high and low levels of CLIC1 mRNA (see Fig. S2) were analyzed by Western blotting altogether with novel cultures to select GSC with low CLIC1 protein content. Western blot analysis of total cell lysates from seven GSC cultures. Membranes were re-probed with α-tubulin antibody after stripping and used as a reference for protein loading (upper panel). Densitometric analysis of CLIC1 levels in the same samples. Data are expressed as mean ± S.D. of CLIC1 densitometric values normalized using respective α-tubulin densitometry (lower panel). GSCs from GBM39 and GBM 44 display low CLIC1 expression as compared to GBM3, 5, 18, and 23. CLIC1 expression in GBM50 was intermediate between these two groups. **B** Growth curves of low- (GBM39) and high- (GBM3, GBM23) CLIC1-expressing GSCs. Cell viability and proliferation measurement was performed by MTT assay (*n* = 3). The growth curves were modeled by non-linear third order polynomial fitting. A significant lower proliferation rate was observed in GSCs isolated from GBM39. **C** Low expressing CLIC1 GSCs retain differentiating ability. Upper pictures: morphological appearance of GBM44 cells grown in stem cell-permissive serum-free medium (STEM, phase contrast microscopic image, bar =10 μm) or after shifting for 2 weeks in 10% FBS-containing medium (DIFF, bar =10 μm). Lower panels: representative western blots of the immunoreactivity for the stem cell marker, Sox2 (left) and for the astrocytic differentiation marker GFAP (right) in GBM44 GSCs and differentiated cell lysates. Membranes were re-probed with β-actin antibody after stripping and used as a reference for protein loading. Histograms report the densitometric analysis of Sox2 and GFAP levels, expressed as mean ± S.D. of densitometric values normalized with β-actin content. GBM44 differentiation is characterized by loss of Sox2 and increased GFAP expression, as occurs in high CLIC1-expressing GBMs. **D** Upper panels: Immunofluorescence analysis of Sox2 expression in GSC spheroids (upper pictures, bar = 200 μm), GSC monolayers (middle pictures, bar = 100 μm) and differentiated GBM cells (lower pictures, bar = 100 μm) in GBM44 culture. Sox2-positive cells are red and nuclei counterstained with DAPI (blue). Lower panel: GFAP-positive cells (red) in GBM44 GSCs grown as monolayer and their differentiated counterpart. Nuclei were counterstained with DAPI (blue). Bar = 200 μm. **E** Antiproliferative activity of Q48, Q54 and metformin in low CLIC1-expressing CLIC1 GSCs. Upper panels: concentration-response curves, obtained from MTT assay after 48 h treatment, in low-expressing GBM37 and GBM44 cultures compared with the average response obtained in high-expressing GBM GSCs. Data represent the pooled mean ± SEM from *N* = 3 independent experiments. Lower Table: mean IC_50_ value (potency) and maximal inhibition (efficacy) reached for each compound obtained from the concentration-response curves. See the text for description

To confirm the role of CLIC1 in biguanides’ antiproliferative activity, we analyzed GSCs derived from GBMs spontaneously expressing this channel at low levels. RNA-seq analysis of different GSC cultures demonstrated that most GBM express CLIC1, with the exception of GBM39, which was characterized by low mRNA content (Fig. S1A). We validated these data, analyzing CLIC1 expression at protein level by Western blotting, and besides GBM39, we identified two other patient-derived cultures (GBM44 and GBM50) showing CLIC1 content lower than the majority of the available GBMs (Fig. 6A).

#### Low-expressing CLIC1 GBM cultures retain in vivo and in vitro features of CLIC1-expressing GSCs

First of all, we compared, by MTT assay, basal proliferation rate of high CLIC1-expressing (GBM3 and GBM23) and spontaneously low CLIC1-expressing (GBM39) GSCs for up to 11 days. Growth curves evidenced that, consistently with the downregulation of CLIC1, GBM39 in vitro proliferation was much slower than GBM3 and GBM23 (Fig. 6B).

However, we have to point out that, not only low CLIC1-expressing GSCs were isolated from patients which developed aggressive GBMs, but also that GBM39 cells retain tumorigenic potential in vivo, similar to high CLIC1-expressing GSCs (mean survival time of orthotopically xenografted mice was 100 days for GBM39 and GBM23, and 120 days for GBM3, Table S1), suggesting that this protein is dispensable for tumor development and progression in vivo. In fact, searching TGCA and CGCA databases to correlate patients’ survival with CLIC1 expression, we did not observe differences in survival rate according to CLIC1 mRNA content (*p* = 0.34 and 0.14, respectively; Fig. S12).

Then we tested whether low CLIC1-expressing GSCs retain stem cell features as high CLIC1-expressing cells. As far as stemness and self-renewal markers, we report that GBM39 cells do not display a different pattern of expression as compared to high CLIC1-expressing tumors (Fig. S13). In particular, RNA-seq analysis demonstrated high expression of CD44, integrin-α6 (ITGA6), Olig2, Sox2, nestin and S100A4 in all 14 GSC cultures analyzed (few exceptions were detected, as expected due to GBM heterogeneity, but they did not correlate with CLIC1 mRNA levels); CD133 was expressed in a subset of GBMs (6/14, including the low CLIC1-expressing GBM39), while CD15 was detected in only two GBMs. From a functional point of view, we demonstrated that GBM44-derived cells retain spherogenesis ability when grown in low attachment conditions, and differentiate into non-stem GBM cells when shifted from stem cell-permissive to FBS-containing medium for 15 days, as reported [27]. In this experimental condition, GSCs changed morphology, down-regulated Sox2 and increased GFAP content, as assessed by Western blotting and IF on GSC spheroids and monolayers (Fig. 6C and D).

These data demonstrate that, although CLIC1 is commonly expressed by GSCs, GBM characterized by low expression can also develop in patients, and GSCs isolated from these tumors retain tumorigenic ability in vivo and the typical in vitro phenotypical and biological features of cancer stem cells. Conversely, the in vitro proliferation rate is significantly hampered by low CLIC1 expression.

#### CLIC1 expression determines biguanide sensitivity in GSCs

To correlate CLIC1 expression with the antitumor response to biguanide derivatives, low CLIC1-expressing GSCs (GBM39 and GBM44) were treated with Q48, Q54 and metformin for 48 h in concentration-response experiments, and cell survival monitored by MTT assay. GBM39 and GBM44 cells display reduced sensitivity to biguanides, as compared to high CLIC1-expressing GSCs. Indeed, at the maximal concentrations tested, 3 mM for Q48 and Q54, and 30 mM for metformin, cell viability was reduced by only − 50, − 55%, and − 49% in GBM39 and − 17, − 40%, and − 50% in GBM44, respectively, as compared to − 81, − 78%, and − 82% as average effect in CLIC1-expressing GBMs (Fig. 6E). Moreover, a 10-fold lower potency was observed calculating IC_50_ of Q48 (from 0.082 mM in high expressing-CLIC1 GBMs to 0.69 mM in GBM39) and metformin (from 9.8 mM to 117.1 mM in GBM39 and 68.7 mM in GBM44). Q54 showed, besides lower efficacy, slightly higher potency in GBM39, as compared to high CLIC1 expressing GBMs (0.12 mM vs. 0.43 mM), while IC_50_ values for Q48 and Q54 in GBM44 cannot be calculated due to the lack of dose-dependent effects (Table S7).

**Fig. 7:**
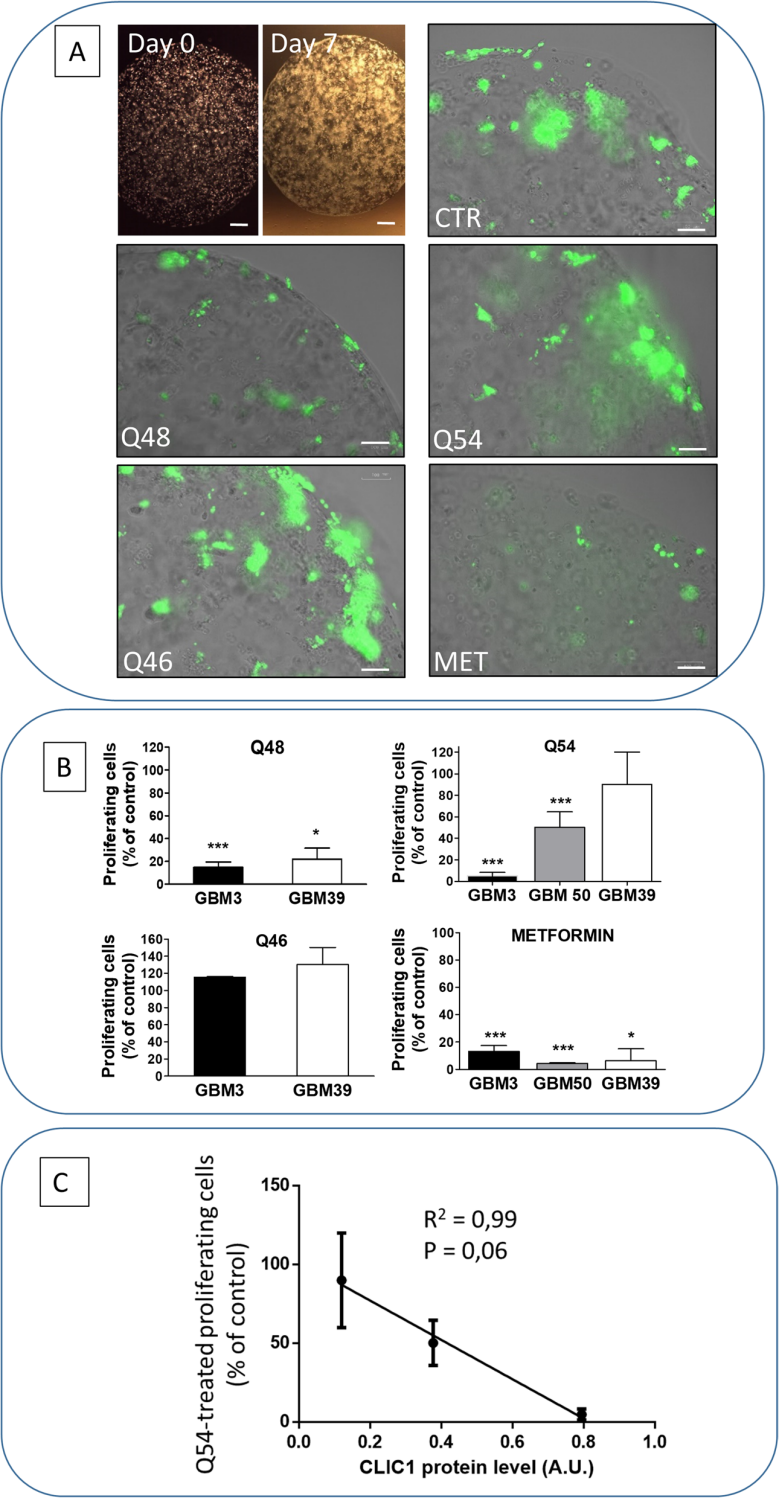
**A** Low CLIC1-expressing GSCs, derived from GBM39, are able to grow as 3D organoids. Up-left pictures: representative images soon after plating (day 0) and after 1 week (day 7); bar = 300 μm. Up-right and following pictures: representative images of GBM39 organoids labeled with 5-EdU (green) to evidence cells in active proliferation. Vehicle-treated cells (CTR) display a lower proliferative rate than high CLIC1-expressing organoids (see Fig. 4A). However, 7-day treatment with Q48 and metformin further reduced proliferating cells. Beside Q46 which was ineffective, as already shown on other parameters, also Q54 was unable to reduce organoid cell proliferation in GBM39. bar = 100 μm. **B** Quantification of Q48, Q54, Q46 (100 μM), and metformin (10 mM) -dependent changes in organoid proliferation rate. Data, obtained using ImageJ software, are reported as % of control values in the graph. Antiproliferative activity is reported comparing the responses in high CLIC1-expressing GSCs (GBM3) and low CLIC1-expressing GSCs (GBM39). Q54 and metformin were also tested on GBM50 which expresses CLIC1 at an intermediate level (see Fig. 5A). While Q48 and metformin reduced proliferation in organoids independently from CLIC1 expression levels, Q54 efficacy was directly proportional to the expression of the channel. *p < 0.05, ***p < 0.001 vs. respective control samples. **C** Linear regression (*R*^2^ = 0.99) correlating CLIC1 expression and Q54 antiproliferative activity in GSC-derived organoids

Low CLIC1-expressing GSCs were able to generate 3D organoids, although showing a lower proliferating cell fraction, confirming that tumor formation may be, at least partially, independent from CLIC1 activity. GSC organoid treatment with Q48, but not Q54, caused a comparable inhibition in GBM3, GBM50, and GBM39, that thus was independent from CLIC1 expression levels (Fig. 7A and B). This unexpected result, as compared with the data obtained in monolayer cultures, could be explained with the longer treatment performed in 3D vs. 2D culture experiments (7 and 2 days, respectively) which may allow the interaction with different molecular targets. Conversely, Q54 effects showed a significant linear relationship with cellular CLIC1 content (Fig. 7A and B), since in GBM3, highly expressing CLIC1, a maximal inhibition was observed (− 95% of proliferating cells vs. control), in GBM50, which displayed an intermediate CLIC1 content, the effect was in-between (− 50%), and in low CLIC1-expressing GBM39, no significant inhibition was detected (Fig. 7C, *R*^2^ = 0.99). Q46 was ineffective in both GBM3 and GBM39, confirming its inability to affect GSC proliferation in both 2D and 3D cultures.

### Biguanide modulation of dihydrofolate reductase (DHFR) activity

To look for possible additional biguanide derivative molecular targets, contributing to antiproliferative activity in GBM with low CLIC1 activity, we measured DHFR activity by a colorimetric assay. Indeed, folate pathway is crucial for cell growth and division and biguanide derivatives have been showed to inhibit DHFR activity [69, 70]. All GSC cultures analyzed express similar levels of DHFR, as shown in RNA-seq analysis, included GBM39, thus excluding differences in sensitivity among different cultures (Fig. S14A). All the compounds tested (Q48 and Q54: 500 μM; metformin: 10 mM,) induced a significant inhibition of DHFR purified enzyme activity, comparable to that caused by the reference drug methotrexate (MTX, 250 nM) (Fig. S14B). However, when tested on intact cells the inhibitory effect of all biguanides was almost completely abolished after 36 h of treatment (a slight, not statistically significant reduction of enzyme activity was observed with Q48 and metformin), while statistical significance (*p* < 0.05) was reached only after 72 h of treatment for Q48 and metformin, although with low efficacy (− 34 and − 28.5%, respectively). Conversely, no effects were observed using Q54 (Fig. S14C).

## Discussion

GBM poor prognosis, in spite of multimodal aggressive treatments, pushes the development of novel therapeutic opportunities as urgent unmet medical need. Metformin displays antitumor activity on GSCs [27, 28], encouraged its possible repositioning for GBM treatment, at least as molecular lead for new derivatives. Several molecular targets have been proposed for metformin (and other biguanides) antitumor effects [28, 33, 71, 72], but the inhibition of CLIC1, whose activity is highly correlated to GSC proliferation [6, 38], is one of the promising options.

Here we tested a small library of novel biguanide derivatives for the ability to affect GSC proliferation, self-renewal and invasiveness with higher potency than metformin, which is active only at concentrations within mM range [27, 28, 71]. Since both linear and cyclic biguanides inhibit CLIC1 activity [38], we analyzed biguanide-based compounds derived from either metformin (linear structure) or cycloguanil (cyclic structure). Linear aryl-biguanides (Q46, Q42, Q48, and Q49), previously studied as trace amine-associated receptor 1 agonists [73], included, as best substitution pattern, lipophilic groups on the aromatic ring, such as 4-Cl (Q42) and 4-CH_3_ (Q49) granting potencies in the μM range, while the polar 4-OCH_3_ group (Q48) produced a negative effect, abolishing the activity. In silico analysis of the pharmacokinetic profile of both linear aryl-biguanides (Q42, Q46, Q48–50) and cycloguanil-like derivatives (Q51-Q54) predicted a slightly better ability to cross the BBB than metformin, but not enough to avoid a carrier-mediated organic cation transport. Noteworthy, in mice, metformin accumulation in CNS was demonstrated, reaching up to 20% of the plasma concentration [74]. Thus, although specific studies should address this issue, we expect the novel compounds to reach CNS with similar (or better) ability than metformin.

Initially, we evaluated the potency of the novel compounds to inhibit GSC proliferation, and the blockade of CLIC1 current as specific molecular signaling mediating this effect, using cells not requiring CLIC1 for proliferation (i.e. non-stem GBM cells and ucMSCs [6]). From this screening, we observed that, within linear biguanides, Q42 and Q50, bearing a lipophilic and electron-withdrawing group (4-Cl and 3-CF_3_) on the phenyl ring, displayed high antiproliferative activity, while the substitution with the apolar and electron-donor 4-CH_3_ (Q49) was responsible for a net decrease of efficacy. In the dihydrotriazine (cyclic biguanides) subset, Q53 and Q52 were, respectively, the 3-Cl and 2-Cl isomers of cycloguanil (4-Cl) which previously demonstrated a mean IC_50_ = 0.18 mM against seven GBMs [38]. Notably, the shift of the chlorine atom from para- (cycloguanil) to meta-position (Q53) was still tolerated, but at the expense of reduced potency, while the ortho-Cl substitution prevented Q52 to acquire an appropriate spatial geometry, likely due to steric hindrance. On the other hand, Q48 and Q54, which are both decorated with a 4-OMe group on the aromatic ring as polar and electron-donor chemical feature (see Fig. 1A), displayed higher potency than metformin as far as GSC antiproliferative activity (IC_50_ = 0.082 and 0.43 mM, respectively, vs. 9.78 mM of metformin), and selectivity as CLIC1 blockers (efficacy of about − 40% cell viability at the highest concentration tested, toward CLIC1-independent cells, as compared to − 80% in GSCs), and were identified as best lead compounds.

Safety of Q48 and Q54 after systemic administration was showed in vivo, demonstrating the absence of zebrafish and chick embryo toxicity after prolonged treatment (up to 5 and 10 days, respectively) using higher concentrations (within the mM range) than antiproliferative IC_50_, thus confirming their low toxicity observed in vitro in non-tumor cell cultures, including ucMSCs and astrocytes. Antitumor activity of Q48 and Q54 also involved the inhibition of GSC spherogenesis, an in vitro index of self-renewal, migration, and invasiveness, with efficacy similar to that of metformin, but a 100-fold higher potency.

The main molecular target of Q48 and Q54 biguanide derivatives was identified in CLIC1, since these compounds directly bind to a recombinant form of the channel, with Kd similar to the IC_50_ observed in the antitumor activity, and act as CLIC1 blockers in GSC whole-cell electrophysiology experiments. The correlation between the inhibition of CLIC1-mediated ion current by Q48, Q54, and the antiproliferative effects was further supported by the observation that Q46 that did not affect GSC proliferation was also inactive as CLIC1 blocker. While these results clearly correlate biguanide ability to bind to CLIC1 and to block CLIC1-mediated current with their antiproliferative activity, it is worth noting that, differently from Q54, the Q48 concentration able to completely hamper CLIC1 activity was higher than antiproliferative IC_50_ (0.5 vs. 0.082 mM), thus implying the possible involvement of adjunctive mechanisms of action.

Then we tested biguanide derivatives activity on GSC proliferation in 3D organoids, and tumor growth in vivo, using the zebrafish model [75]. When grown in Matrigel™ droplets, GSCs develop a tissue-like structure characterized by an external layer of fast-proliferating cells expressing stem cell markers, and internal layers of slow-proliferating cells with differentiated phenotype (i.e. expression of β-III tubulin and GFAP), nicely reproducing GBM growth. Seven-day treatment with Q48 and Q54 (but not Q46) inhibited GSC organoid growth with efficacy similar to metformin but with higher potency. In vivo*,* Q54 significantly reduced the growth of GBM3 GSCs, xenotransplanted in zebrafish hindbrain, while Q46 was ineffective, as already shown in vitro. Conversely, Q48 which was highly active in vitro, caused a modest inhibition of GSC growth in vivo, which did not reach the statistical significance. Presently, we do not have a definitive explanation for the low activity of Q48 in vivo, but a possibility could reside in the animal model used (zebrafish) which requires testing molecules to be added to the fishes’ culture water. In particular, being Q48 much less hydrosoluble than Q54, it might not be able to reach effective concentrations in animal brain.

Interestingly, in both 2D and 3D cultures, Q48 and Q54 reduced proliferating cell number mainly acting on the GSC component (i.e. Sox^+^ cells). Moreover, also other relevant stem-like features strictly related to GBM relapse [76], including self-renewal, migration and extracellular matrix invasion, were selectively inhibited by these molecules, further supporting their potential efficacy also in more complex settings.

One of the main issues for clinical translation of preclinical studies using metformin and derivatives is in vivo safety of the corresponding antitumor concentrations active in vitro. The effective antiproliferative concentration we identified in the in vitro model in which short-term treatments (48-72 h) are used, is in the mM range, accordingly to most studies reporting antitumor activity of metformin in vitro. Plasma concentrations corresponding to the standard treatment in diabetes patients (1.5-2 g/day = ≈30 mg/kg) correspond to about 10–30 μM [77]. However, considering the peculiar pharmacokinetics of metformin, plasma concentration is not a completely reliable parameter, since the molecule is highly water-soluble and its plasma persistence is reduced while it progressively accumulates in tissues [78]. Tissue accumulation, involving red blood cells and liver among others, is amplified in tumors, where OCT1 and other transporters are overexpressed [79]. A similar discrepancy is detected also in experimental animal studies. Daily oral administration of metformin to mice at a dose of 250 mg/kg (about 10-fold higher than the human dose on a mg/kg basis) for 2 weeks leads to a 5 μM plasma concentrations, comparable to the levels obtained during diabetes treatment, while tumor-bearing mice daily treated with 350 mg/kg metformin *p.o.* showed high tumor and liver accumulation (concentration around 1500 and 200 μM, respectively) without evident systemic side effects [80]. In line with these observations, data in literature and our previous studies demonstrated that that prolonged exposure (15 days) of GBM cells to metformin showed antiproliferative activity after treatment with only 10 μM [6]. Thus short-term in vitro treatments need to use high concentrations to mimic this accumulation process occurring after prolonged administration, as likely occurs when metformin will be used as adjuvant antitumor therapy. As far as the novel compounds, in this study we obtained indications of a substantial safety of the efficacious concentrations. In fact, both Q48 and Q54 are effective at lower concentrations than metformin, that are close to the metformin plasma levels identified in diabetic patients (82 and 430 vs. 10–30 μM). Thus concentrations within the micromolar range, which display antitumor effects in GSCs, are safely reachable in humans. In fact, we did not observe in vivo toxicity when Q48 and Q54 are used at higher concentrations (20–100 fold) than the calculated IC_50_ for the antiproliferative activity in vitro, even after prolonged treatments (10 days in ovo and 5 days in zebrafish embryos). Although novel biguanides Q48 and Q54 are preclinical drug candidates, therefore still lack safety and pharmacokinetic data in humans, preliminary toxicological assessment reasonably predicts their low toxicity and the potential translational relevance if used at the efficacious concentrations identified in this study.

Altogether these data provide relevant pieces of information: (i) CLIC1 inhibition reduces GSC proliferation, self-renewal, migration, and invasiveness; (ii) the antiproliferative activity of biguanides (including the novel derivatives characterized in this study) is mainly mediated by CLIC1 inhibition, as shown by the minimal inhibition of viability observed in ucMSCs, which do not require CLIC1 activity to proliferate, or in GSCs interfered for CLIC1 expression; (iii) metformin antitumor activity can be improved, as demonstrated by the novel compounds tested, in particular as far as potency, without losing the safety of the antidiabetic drug, as shown by the low toxicity in normal astrocytes and ucMSCs, or when systemically administered to whole organisms; (iv) antitumor effects exerted in 3D GSC organoids and in vivo in zebrafish are highly suggestive of efficacy also in more complex systems. These latter data also provide evidence that, although unable to freely diffuse through membranes, these compounds are able to be vehiculated inside tissues, likely by transport systems. However, similarly to metformin, the physical-chemical properties of the novel biguanide derivatives allow their ionization at physiological pH in tissues, a molecular condition required for an effective interaction with CLIC1. In fact, in the only model available, basing on single-amino acid mutations in the putative pore region, it was proposed that biguanides might affect channel activity by interacting with negative charged amino acids [6]. Further studies are required to directly characterize this aspect.

Current oncological drug development, in the view of precision medicine, is focused on the identification of molecular drug targets to improve pharmacological activity and reduce systemic toxic effects. Thus, analysis of drug target gene expression in individual patients is a mandatory requirement. CLIC1, highly expressed in most GBMs, acts as a main regulator of growth rate (see [1, 8], and this study). Here, we describe for the first time the distribution of CLIC1 in discrete human GBM cells. The presence of intense staining in cells localized near vessels, resembling infiltrating cells, supports not only the well-known role of this ion channel in cell division and motility [7], but also the importance to develop a pharmacological strategy to inhibit CLIC1, as proposed in this study. However, we identified few human GBMs (namely, GBM39, GBM44, and GBM50) natively expressing this channel at low levels, which could represent a subset of tumors in which CLIC1 activity is bypassed by different intracellular signaling. Interestingly, these tumors in patients, and the relative GSCs isolated in vitro, display similar features than CLIC1 high-expressing counterparts. In fact, the analysis of the expression of several stem-related markers (CD44, CD133, CD15, ITAG6, Olig2, Sox2, nestin and S100), while showing the expected heterogeneity in different GBMs, did not detect a specific pattern for low CLIC1 expressing GBMs. Moreover, in 2D and 3D cultures these cells behave similarly to GSCs with high CLIC1 activity (for example as far as spherogenesis, differentiation ability, organoid development). Thus, while CLIC1 functional expression enhances GSC proliferation and tumorigenesis, its activity can be circumvented in some GBMs through the activation of alternative intracellular pathways allowing tumor development and progression also when the channel activity is negligible, as also shown by the lack of differences in patients’ survival according to CLIC1 expression (see Fig. S12). However, in these tumors antitumor activity of metformin and novel biguanides is significantly impaired. Conversely, in tumors which express CLIC1 and display high ion channel activity, its blockade is deleterious for GSC growth and invasiveness, highlighting that, in the majority of GBMs, CLIC1 targeting might represent a powerful therapeutic option.

We propose that the identification of CLIC1-dependent tumors may allow the selection for GBM (and also other tumor types) patients likely receiving benefit from biguanide treatment.

However, the study of low CLIC1-expressing GBMs allowed us to check the specificity of the novel biguanides as CLIC1 blockers. In 2D cultures of CLIC1 low-expressing GSCs, a significant reduction in potency and efficacy of Q48 and Q54 (and metformin) was detected. It is worth noting that the novel biguanides retained, although impaired, antiproliferative activity, likely because GSCs from these GBMs still express low levels of CLIC1, which represent the expected molecular target of these compounds. Similarly, lack of biguanide efficacy was also observed in differentiated GBM cells and ucMSCs, in which no CLIC1 activity can be recorded [6], or in GSCs in which CLIC1 was down-regulated. Interestingly, in 3D cultures metformin and Q48 produced a significant antitumor activity independently from CLIC1 expression in GBM39 GSCs, while Q54 efficacy was completely dependent on CLIC1 content. This discrepancy may have different explanations: it may rely on the experimental protocols used (i.e. 3D cultures were treated for 7 days while the 2D only for 2 days), since metformin and, likely, other linear compounds accumulate within tumor tissues and cells after prolonged treatments [78], displaying increased antitumor efficacy proportional with duration of treatment [6]); alternatively this observation could highlight the hit of additional molecular GSC targets involved in the control of proliferation. To address this issue, we assessed the inhibitory activity of biguanides on DHFR, as previously reported [69]. Both metformin and the novel derivatives reduced purified enzyme activity, but, when the treatment was performed in living cells, only Q48 and metformin, but not Q54, caused a moderate enzyme inhibition after 72 h of treatment. Previously, Q54 was proven to display low toxicity against human cell lines when tested as DHFR inhibitor [70], due to limited ability to access to the intracellular compartment. Herein, we observed that DHFR activity inhibition by biguanides was time-dependent and likely related to an uneven internalization of the compounds, that was less efficient for the cyclic derivative Q54. Thus, the prolonged treatment of 3D cultures allowed a significant cellular internalization of metformin and Q48 (but not of Q54), resulting in a significant inhibition of GSC growth also in low CLIC1-expressing GBMs. It has to be remarked that according to the proposed model [6], CLIC1 binding site is located on the external side of plasma-membrane thus being easily accessible for ionized compounds. Moreover, the involvement of other intracellular pathways in Q48 activity is also conceivable by the discrepancy between the antiproliferative IC_50_ and the concentration required to completely block CLIC1-mediated current (0.082 mM vs. 0.5 mM).

## Conclusions

We show that, similarly to metformin, Q48 and Q54 affect GSC proliferation, self-renewal, migration, and invasion, mainly acting via CLIC1 inhibition. Antitumor effects were observed in both 2D and 3D models, and, for Q54, in vivo in the zebrafish model. Importantly, our data, considering the low toxicity for normal cells and the significant higher potency than metformin, support the possible clinical translation of Q48 and Q54, or of derivatives obtained from these lead compounds. Moreover, we propose that CLIC1 is a major determinant of cancer stem cell activity, at least in GBM, controlling not only cell proliferation but also invasiveness and, more in general, tumorigenesis, and that antitumor activity of biguanide-based compounds requires its inhibition, among all the different intracellular mechanisms that these drugs can affect. Thus CLIC1 expression and activity represents a relevant biomarker which have to be considered to predict GBM responsiveness to these novel biguanide derivatives. In fact, we also identified a subset of GBM displaying a CLIC1-independent growth, in which CLIC1 activity is likely bypassed by the activity of different intracellular signaling. In these tumors, biguanides are less effective although adjunctive mechanisms, including the ability to inhibit DHFR activity, may cooperate to induce antiproliferative effects. However, this effect may be hampered according to the compound analyzed due to the uneven intracellular permeation of these compounds within GSCs, being prone to ionization due to their strongly basic nature. On the other hand, their hydrosolubility is essential for an effective interaction between these molecules and CLIC1 channel pore. Hence, we believe that Q48 and Q54 may represent relevant leads to develop novel, powerful and selective treatments for GBM and other CLIC1-dependent tumors, in a precision medicine oriented approach.

## Supplementary Information

**Additional file 1: Figure S1.** Immunohistochemical distribution of CLIC1 in GBM. **Figure S2.** A) CLIC1 expression in GSCs isolated from 14 human GBMs, evaluated by RNA-seq, and expressed as counts per million reads mapped (CPM). Only GBM 39 (arrow) displayed low CLIC1 mRNA content. The Table reports the corresponding codes of the GSC cultures used in this study and the RNA-seq data deposited at NCBI Geo data set. B) Upper panel: CLIC1 expression evaluated by WB, in total cell lysates from selected GSC cultures. Membranes were re-probed with α-tubulin antibody after stripping and used as a reference for protein loading. Lower panel: Histograms report CLIC1/α-tubulin ratio of densitometric values and expressed in arbitrary units (A.U.) as mean ± S.D. **Figure S3.** Dose-response curves of novel biguanide derivatives and metformin on individual GSC cultures. The average response is reported in the Fig. 1A of the manuscript. **Figure S4.** Dose-response curves of novel biguanide derivatives and metformin on individual non-stem differentiated GBM cell cultures (GBM D). The average response is reported in the Fig. 1B of the manuscript. **Figure S5.** Dose-response curves of metformin and novel biguanide derivatives on individual ucMSC cultures. The average response is reported in the Fig. 1C of the manuscript. **Figure S6.** Kaplan-Mayer curves of Q48, Q54, and metformin (MET) depicting the effect of supramaximal concentration of these compounds on zebrafish embryos survival. Limited toxicity, not different from controls, was observed for all the compounds up to 5 days of treatment. Experiments were repeated twice, *n* = 20 per experimental group. Q48: log rank test for trend *p* = 0.62; Q54: log rank test for trend *p* = 0.64; metformin: log rank test for trend *p* = 0.38. **Figure S7.** Effect of different doses of metformin and Q54 on chick embryo survival after 10 days of incubation. Experiments were performed by Inovotion (La Tronche, France). No toxicity was observed for these compounds up to 3 mM. Experiments were repeated twice, *n* = 18 per experimental group. **Figure S8.** A. Dose-response curves of Q46, Q48, Q54 and metformin on rat astrocyte cultures. Limited toxicity is observed for all the novel compounds. Only metformin reduced astrocyte viability (− 50%) at the highest concentration tested (30 mM). Data are expressed as average of experiments preformed in quadruplicate and repeated twice. B. Table reports IC50 values in non-malignant rat astrocytes and GSCs and the calculated selectivity indices for each compound. According to the “selectivity criteria” all biguanides are considered selective compounds against GSCs (selectivity index > 10) (see reference [62]). **Figure S9.** A. CLIC1 expression in GBM19 GSCs carrying siRNA for both Luciferase (siLuc, silencing control) and CLIC1 (siCLIC1). B. Cell proliferation of GBM19 siLuc and GBM19 siCLIC1, treated with Q48 and Q54 (100 μM) for 48 h and evaluated by counting live cells. Data represent the mean ± S.E.M. ***p < 0.001 vs. respective control (CTR). **Figure S10.** Upper panels: Western blot depicting phospho-ERK1/2 (pERK) levels in GSC cultures in control conditions (CTR), after 5 min stimulation with bFGF (40 ng/ml) and after treatment of bFGF-stimulated cells with with Q48 (100 μM), Q54 and Q46 (300 μM), and metformin (10 mM) for 24 h. α-tubulin was used as loading reference. M.W. = molecular weight markers. Lower panels: densitometric analysis of pERK normalized for α-tubulin levels, * = *p* < 0.05; ** = *p* < 0.01. **Figure S11.** Comparison of CD44 (A) and MAP2 (B) mRNA expression in GBM3 GSCs cultivated as 2D monolayer or grown as 3D organoids, for 15, 21, and 30 days. Data are obtained by quantitative RT-PCR experiments. **Figure S12.** Kaplan-Meier analysis of the relationship between overall survival and CLIC1 expression. The statistical difference between the curves is measured by log-rank test. The prognostic effect of CLIC1 mRNA level in GBM according to The Cancer Genome Atlas (TGCA) databasea nalyzed using the GEPIA (Gene Expression Profiling Interactive Analysis database) software (A) and Chinese Glioma Genome Atlas (CGCA) (B). TPM: transcripts of per million; n(high): samples with expression level higher than the median of TPM; n(low): samples with expression level lower than the median of TPM. **Figure S13.** Expression stem cell-related markers (CD44, CD133, CD15, integrin-α6 ITGA6, Olig2, SOX2, S100A4, and nestin) in GSCs isolated from 14 human GBMs, evaluated by RNA-seq, and expressed as counts per million reads mapped (CPM). RNA-seq have been data deposited at NCBI Geo data set (see Figure S2 for the relative codes). Low CLIC1-expressing cells (GBM39) do not display differences as compared to high expressing GBMs. **Figure S14.** A) Dihydrofolate reductase (DHFR) expression in 14 human GSC cultures evaluated by RNA-seq, and expressed as counts per million reads mapped (CPM). Comparable expression was detected in cells derived from all the GBM analyzed. RNA-seq have been data deposited at NCBI Geo data set (see Figure S2 for the relative codes). B) Effect of Q48, Q54, Q46 and metformin (MET) on DHFR activity, incubating the compounds with purified enzyme. Methotrexate (MTX) was used as positive control. Q48, Q54, and MET, but not Q46, inhibited DHFR activity with an efficacy comparable to MTX. * *p* < 0.05. C) Effect of Q48, Q54, and metformin, on DHFR activity in living cells. Q48 and metformin, but not Q54, caused a moderate inhibition of enzyme activity in a time-dependent manner. * *p* < 0.05. **Table S1.** Patients’ and tumors’ characteristics. **Table S2.** Elemental analysis of biguanide derivatives. **Table S3.** Acidity constant (pKa) and distribution constant (LogD) at pH 7 of the novel biguanide derivatives and reference drugs (metformin and cycloguanil). **Table S4.** Statistical analysis of the effects of novel biguanide derivatives and metformin on GSCs. **Table S5.** Statistical analysis of the effects of novel biguanide derivatives and metformin on differentiated non-stem GBM cells. **Table S6.** Statistical analysis of the effects of novel biguanide derivatives and metformin on ucMSCs. **Table S7.** Statistical analysis of the effects of novel biguanides on low CLIC1-expressing GSCs (GBM39 and GBM44) and the average results from high CLIC1-expressing GSCs (GBM3, 5, 19, 23, and 37).

## Data Availability

GSC RNA-seq data have been loaded as NCBI Geo data set (for code correspondence see Fig. S2). All other original data generated during this study are included in the manuscript.

## Abbreviations

2D: Two-dimensional
3D: Three-dimensional
BPBS: Phosphate saline buffer containing 0.1% Triton X-100
CLIC1: Chloride intracellular channel 1
DHFR: Dihydrofolate reductase
Dpf: Days post-fertilization
Hpf: Hours post-fertilization
GBM: Glioblastoma
GFAP: Glial fibrillary acidic protein
GSC: Glioblastoma stem cells
IF: Immunofluorescence
MTT: 3-(4,5-dimethylthiazol-2-yl)-2,5-diphenyltetrazolium bromide
MTX: Methotrexate
NOD/SCID mice: Non-obese diabetic severe combined immunodeficient mice
Olig2: Oligodendrocyte transcription factor 2
PBS: Phosphate saline buffer 0.01 M
PFA: Paraformaldehyde
ROS: Reactive oxygen species
RPLP0: Ribosomal protein large P0
RT: Room temperature
Sox2: Sex determining region Y-box 2
tmCLIC1: Transmembrane isoform of chloride intracellular channel 1
UC: Umbilical cord
ucMSC: Umbilical cord-derived mesenchymal stem cells
WB: Western blot
