# Supplementary material for: Chloride intracellular channel 1 activity is not required for glioblastoma development but its inhibition dictates glioma stem cell responsivity to novel biguanide derivatives
Source: J Exp Clin Cancer Res. 2022 Feb 8;41:53. doi: 10.1186/s13046-021-02213-0 (PMC8822754; doi:10.1186/s13046-021-02213-0)
Supplement: Supplementary file 1 — Additional file 1: Figure S1. Immunohistochemical distribution of CLIC1 in GBM. Figure S2. A) CLIC1 expression in GSCs isolated from 14 human GBMs, evaluated by RNA-seq, and expressed as counts per million reads mapped (CPM). Only GBM 39 (arrow) displayed low CLIC1 mRNA content. The Table reports the corresponding codes of the GSC cultures used in this study and the RNA-seq data deposited at NCBI Geo data set. B) Upper panel: CLIC1 expression evaluated by WB, in total cell lysates from selected GSC cultures. Membranes were re-probed with α-tubulin antibody after stripping and used as a reference for protein loading. Lower panel: Histograms report CLIC1/α-tubulin ratio of densitometric values and expressed in arbitrary units (A.U.) as mean ± S.D. Figure S3. Dose-response curves of novel biguanide derivatives and metformin on individual GSC cultures. The average response is reported in the Fig. 1A of the manuscript. Figure S4. Dose-response curves of novel biguanide derivatives and metformin on individual non-stem differentiated GBM cell cultures (GBM D). The average response is reported in the Fig. 1B of the manuscript. Figure S5. Dose-response curves of metformin and novel biguanide derivatives on individual ucMSC cultures. The average response is reported in the Fig. 1C of the manuscript. Figure S6. Kaplan-Mayer curves of Q48, Q54, and metformin (MET) depicting the effect of supramaximal concentration of these compounds on zebrafish embryos survival. Limited toxicity, not different from controls, was observed for all the compounds up to 5 days of treatment. Experiments were repeated twice, n = 20 per experimental group. Q48: log rank test for trend p = 0.62; Q54: log rank test for trend p = 0.64; metformin: log rank test for trend p = 0.38. Figure S7. Effect of different doses of metformin and Q54 on chick embryo survival after 10 days of incubation. Experiments were performed by Inovotion (La Tronche, France). No toxicity was observed for these compounds up to 3 m [file 13046_2021_2213_MOESM1_ESM.pdf]

SUPPLEMENTARY MATERIAL TO

CHLORIDE INTRACELLULAR CHANNEL 1 ACTIVITY IS NOT REQUIRED FOR GLIOBLASTOMA DEVELOPMENT BUT ITS INHIBITION DICTATES GLIOMA STEM CELL RESPONSIVITY TO NOVEL BIGUANIDE DERIVATIVES

Federica Barbieri[1*], Alessia Graziana Bosio[1*], Alessandra Pattarozzi[1], Michele Tonelli[2], Adriana Bajetto[1], Ivan Verduci[3], Francesca Cianci[3], Gaetano Cannavale[3], Luca M. G. Palloni[3], Valeria Francesconi[2], Stefano Thellung[1], Pietro Fiaschi[4,5], Samanta Mazzetti[6], Silvia Schenone[2], Beatrice Balboni[7,8], Stefania Girotto[7], Paolo Malatesta[4], Antonio Daga[4], Gianluigi Zona[4,5], Michele Mazzanti[3+], and Tullio Florio[1, 4+]

[1] *Sezione di Farmacologia, Dipartimento di Medicina Interna, Università di Genova, 16132, Genova, Italy*

[2] *Dipartimento di Farmacia, Università di Genova, 16132 Genova, Italy*

[3] *Dipartimento di Bioscienze, Università degli Studi di Milano, 20133 Milano, Italy*

[4] *IRCCS, Ospedale Policlinico San Martino, 16132, Genova, Italy*

[5] *Dipartimento di Neuroscienze, Riabilitazione, Oftalmologia, Genetica e Scienze Materno-Infantili, Università di Genova, 16132, Genova, Italy*

[6] *Fondazione Grigioni per il Morbo di Parkinson, 20135, Milano, Italy*

[7] *Computational and Chemical Biology, Fondazione Istituto Italiano di Tecnologia, 16163 Genova, Italy*

[8] *Department of Pharmacy and Biotechnology, University of Bologna, 40126 Bologna, Italy*

* Equally contributed

+ Correspondence to:

Tullio Florio: Tullio.florio@unige.it

Michele Mazzanti: michele.mazzanti@unimi.it

This PDF file includes:

1) **Supplementary Figures S1 – S14**

2) **Supplementary Tables S1 – S7**

SUPPLEMENTARY FIGURES

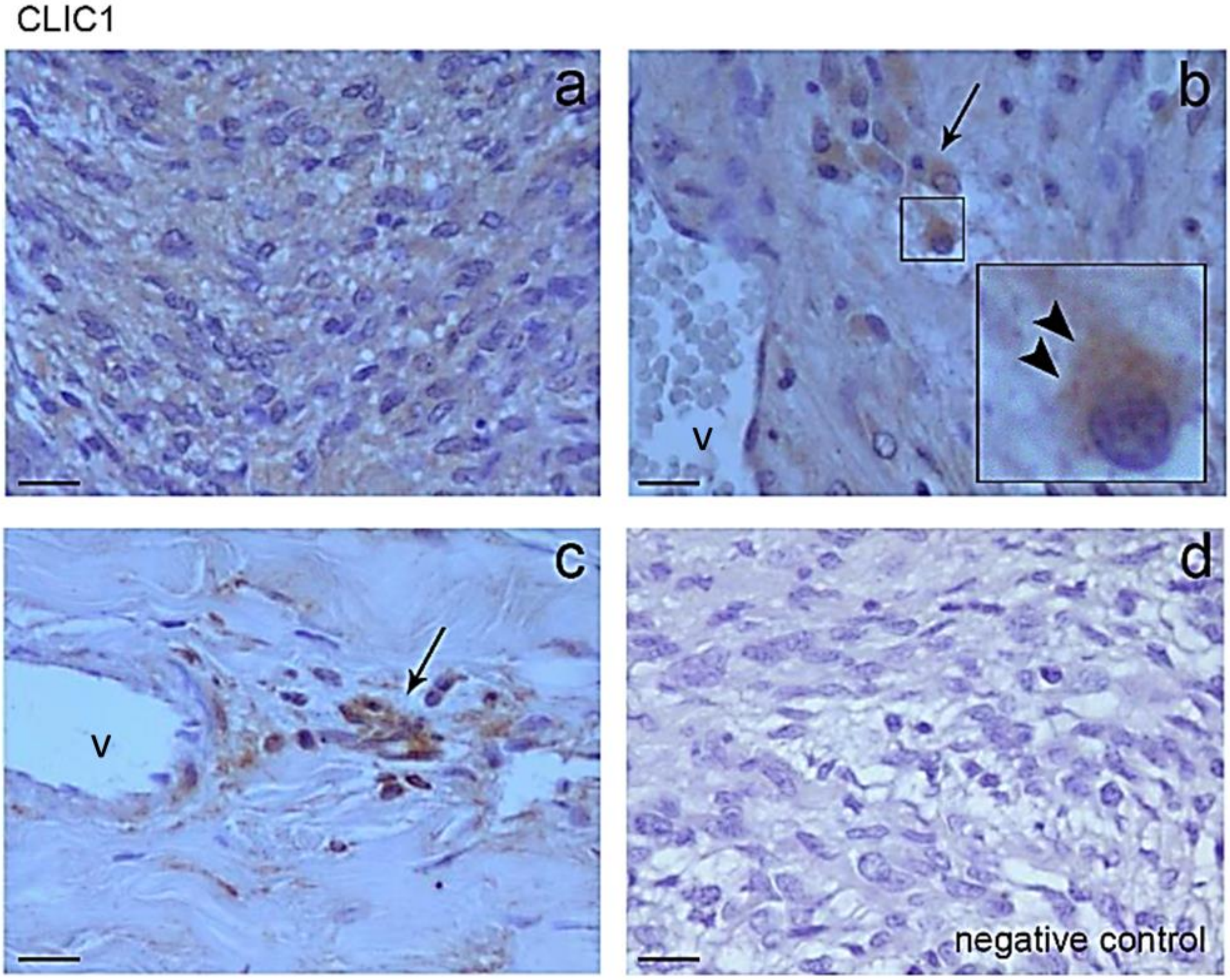

FIGURE S1

Distribution of CLIC1 in GBM

(a) GBM solid tumor cells CLIC1 staining is diffuse and uniform.

(b-c) CLIC1 intensely positive cells are visible near vessels (arrows), and the staining is dot-like and localized especially on plasma membrane (arrows-heads).

(d) Negative controls.

V: vessel.

Scale bar = 40 μm

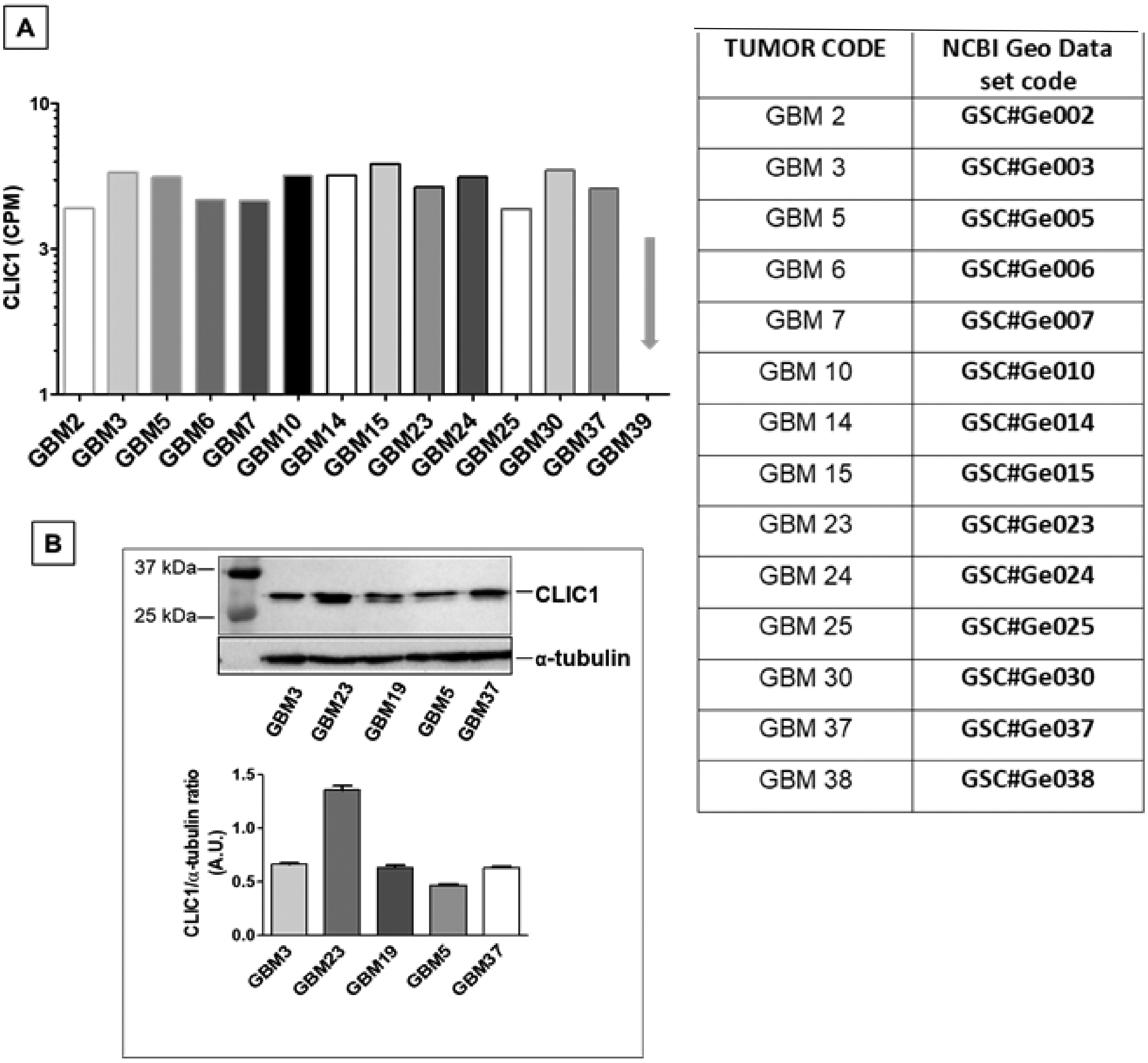

TUMOR CODE	NCBI Geo Data set code
GBM 2	GSC#Ge002
GBM 3	GSC#Ge003
GBM 5	GSC#Ge005
GBM 6	GSC#Ge006
GBM 7	GSC#Ge007
GBM 10	GSC#Ge010
GBM 14	GSC#Ge014
GBM 15	GSC#Ge015
GBM 23	GSC#Ge023
GBM 24	GSC#Ge024
GBM 25	GSC#Ge025
GBM 30	GSC#Ge030
GBM 37	GSC#Ge037
GBM 38	GSC#Ge038

FIGURE S2

A) CLIC1 expression in GSCs isolated from 14 human GBMs, evaluated by RNA-seq, and expressed as counts per million reads mapped (CPM). Only GBM 39 (arrow) displayed low CLIC1 mRNA content. The Table reports the corresponding codes of the GSC cultures used in this study and the RNA-seq data deposited at NCBI Geo data set.

B) **Upper panel**: CLIC1 expression evaluated by WB, in total cell lysates from selected GSC cultures. Membranes were re-probed with α-tubulin antibody after stripping and used as a reference for protein loading.

Lower panel: Histograms report CLIC1/α-tubulin ratio of densitometric values and expressed in arbitrary units (A.U.) as mean ± S.D.

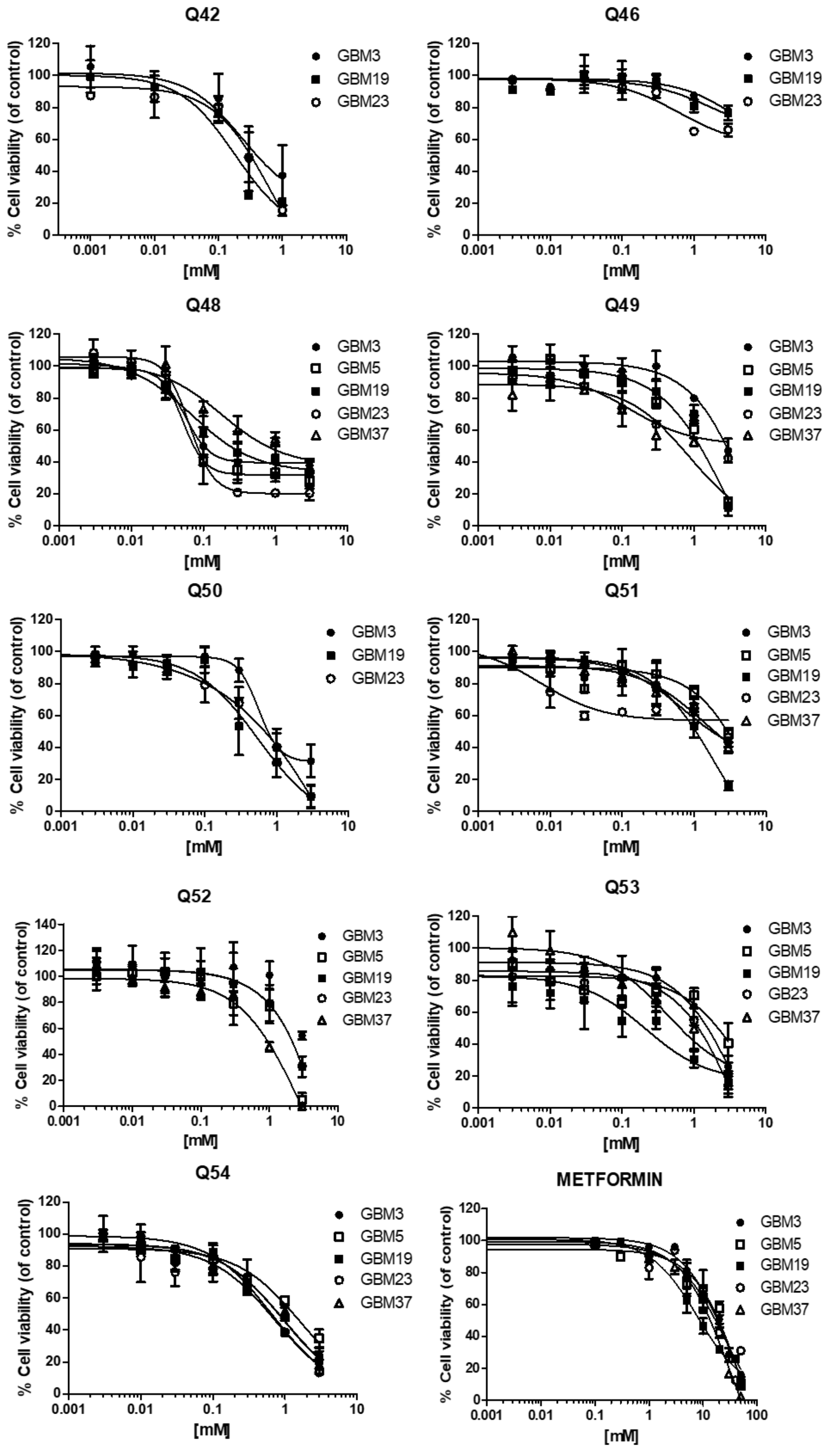

FIGURE S3

Dose-response curves of novel biguanide derivatives and metformin on individual GSC cultures. The average response is reported in the Figure 1A of the manuscript.

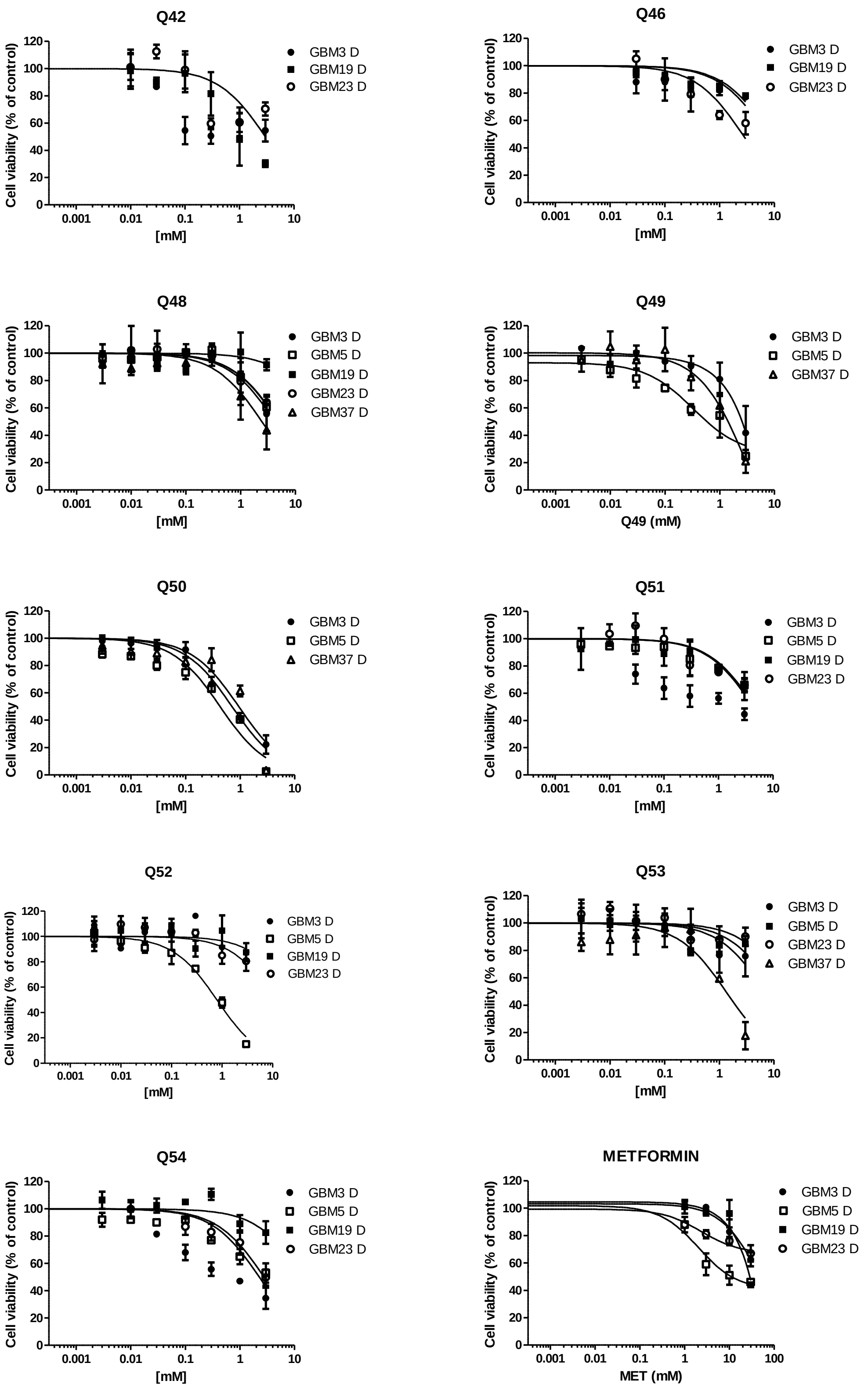

FIGURE S4

Dose-response curves of novel biguanide derivatives and metformin on individual non-stem differentiated GBM cell cultures (GBM D). The average response is reported in the Figure 1B of the manuscript.

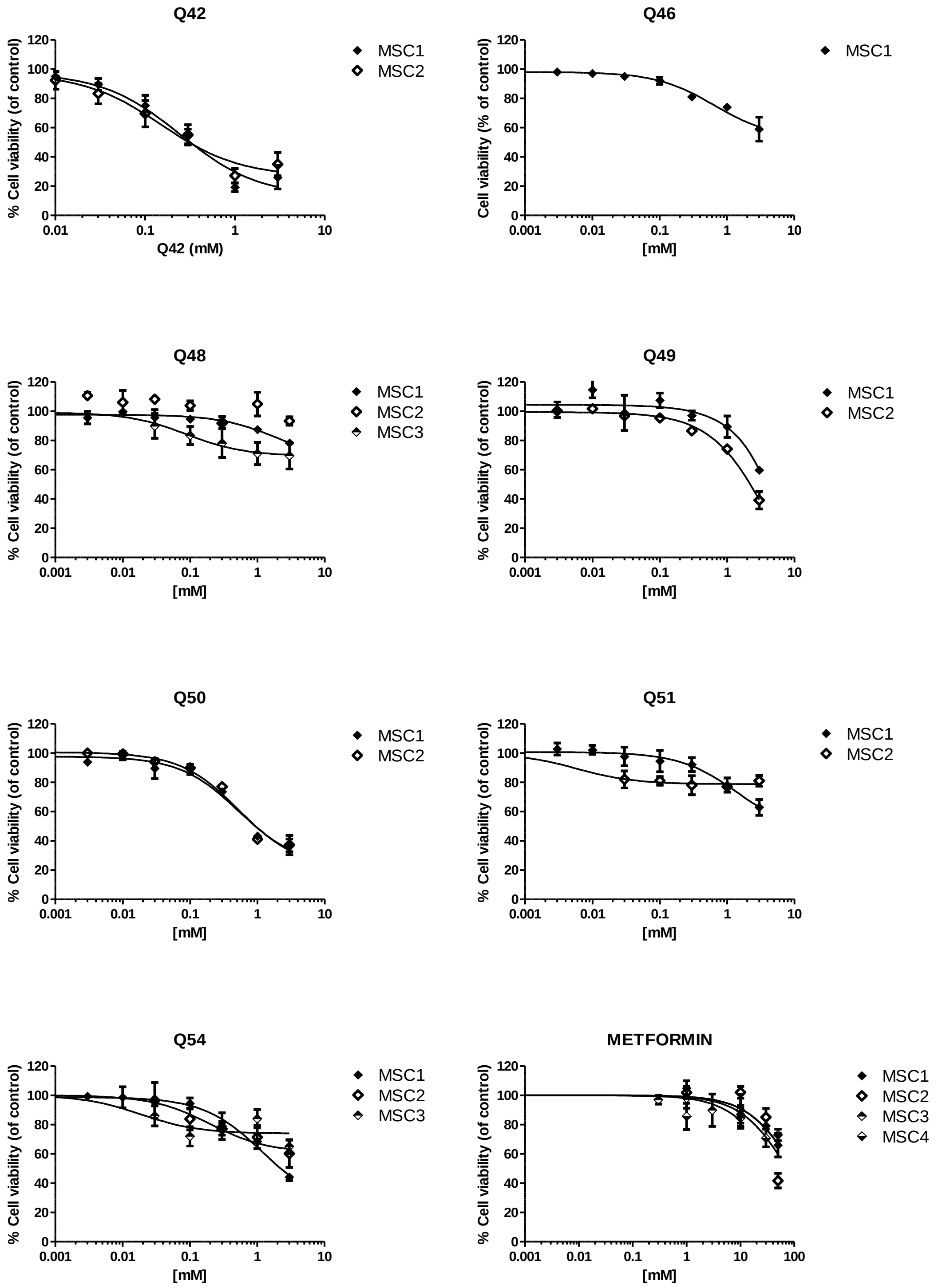

FIGURE S5

Dose-response curves of metformin and novel biguanide derivatives on individual ucMSC cultures. The average response is reported in the Figure 1C of the manuscript.

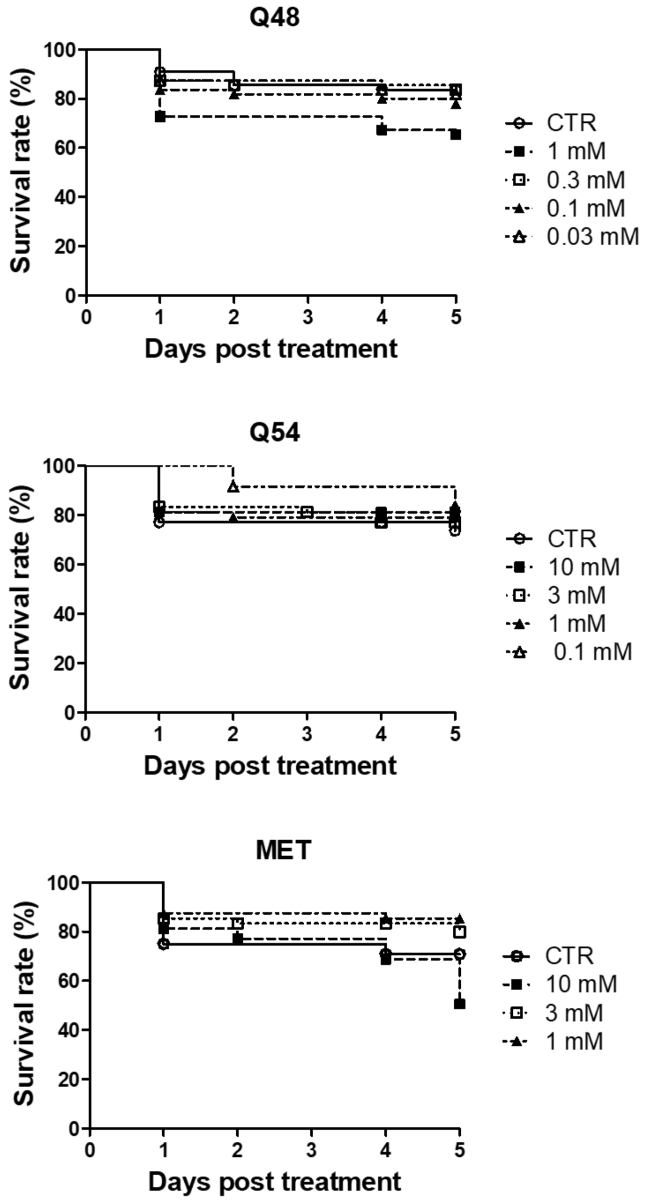

FIGURE S6

Kaplan-Mayer curves of Q48, Q54, and metformin (MET) depicting the effect of supramaximal concentration of these compounds on zebrafish embryos survival. Limited toxicity, not different from controls, was observed for all the compounds up to 5 days of treatment. Experiments were repeated twice, n = 20 per experimental group. Q48: log rank test for trend p=0.62; Q54: log rank test for trend p=0.64; metformin: log rank test for trend p=0.38.

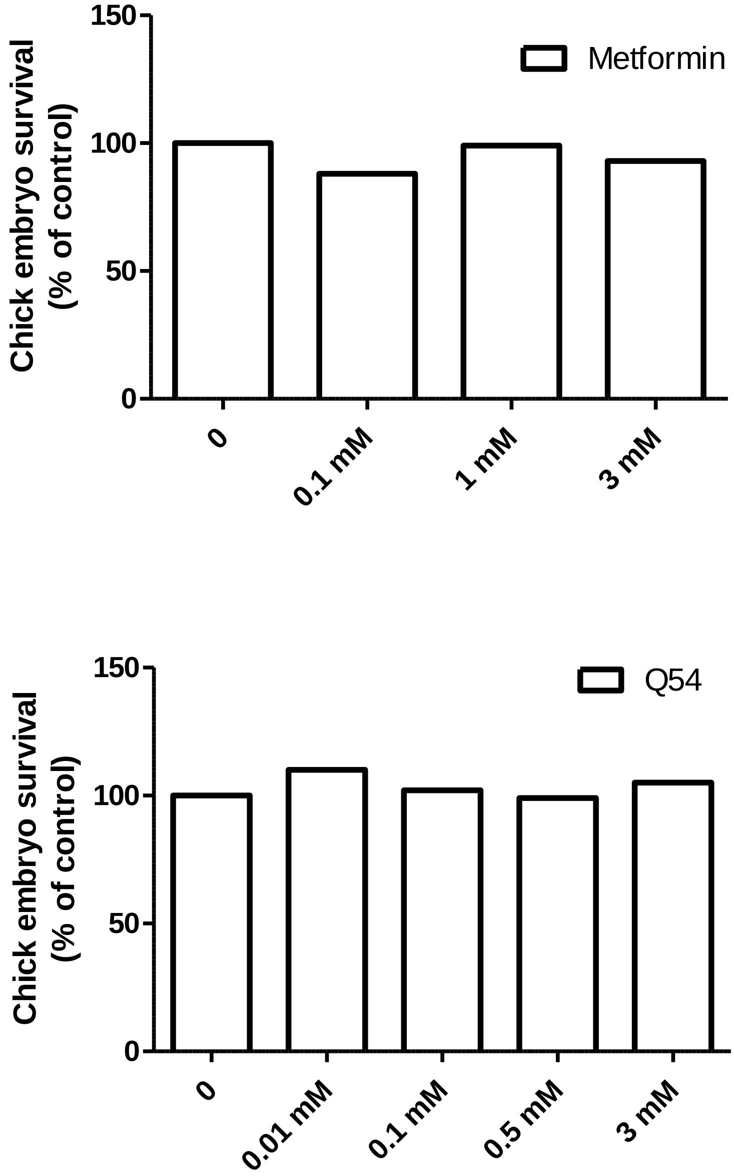

FIGURE S7

Effect of different doses of metformin and Q54 on chick embryo survival after 10 days of incubation. Experiments were performed by Inovotion (La Tronche, France). No toxicity was observed for these compounds up to 3 mM. Experiments were repeated twice, n = 18 per experimental group.

A

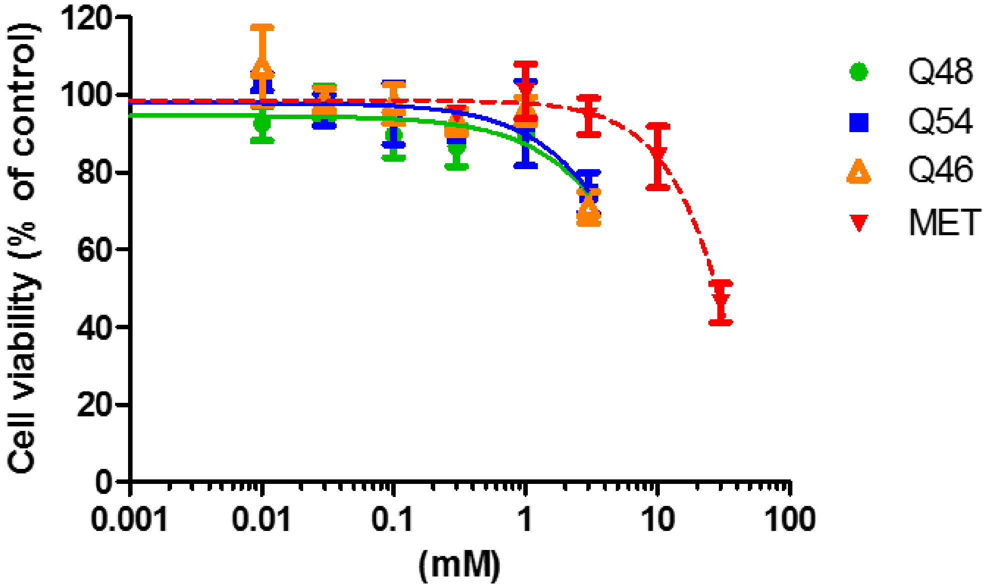

B

Compound#	IC_{50} (mM) Rat astrocytes	IC_{50} (mM) Human GSCs	Selectivity index $\left(SI = \frac{IC_{50}\ no\ cancer\ cells}{IC_{50}\ cancer\ cells} \right)$
Q48	20.19	0.082	246,21
Q54	22.35	0.43	51.97
Q46	n.r.°	n.r.°	-
MET	9.8	106	10,81

°n.r.= not reached

FIGURE S8

A. Dose-response curves of Q46, Q48, Q54 and metformin on rat astrocyte cultures. Limited toxicity is observed for all the novel compounds. Only metformin reduced astrocyte viability (-50%) at the higher concentration tested (30 mM). Data are expressed as average of experiments preformed in quadruplicate and repeated twice.

B. Table reports IC_{50} values in non-malignant rat astrocytes and GSCs and the calculated selectivity indices for each compound. According to the "selectivity criteria" all biguanides are considered selective compounds against GSCs (selectivity index >10) (see reference 62).

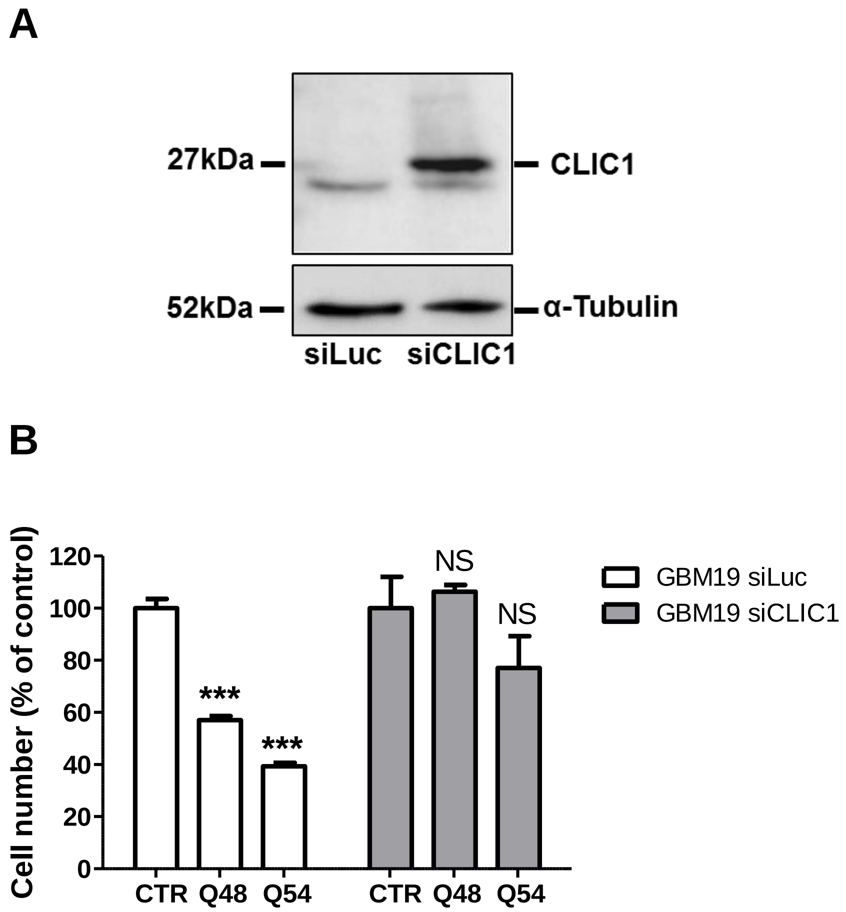

FIGURE S9

A. CLIC1 expression in GBM19 GSCs carrying siRNA for both Luciferase (siLuc, silencing control) and CLIC1 (siCLIC1).
B. Cell proliferation of GBM19 siLuc and GBM19 siCLIC1, treated with Q48 and Q54 (100μM) for 48h and evaluated by counting live cells. Data represent the mean ± S.E.M..

   $^{***}p < 0.001$ *vs.* respective control (CTR).

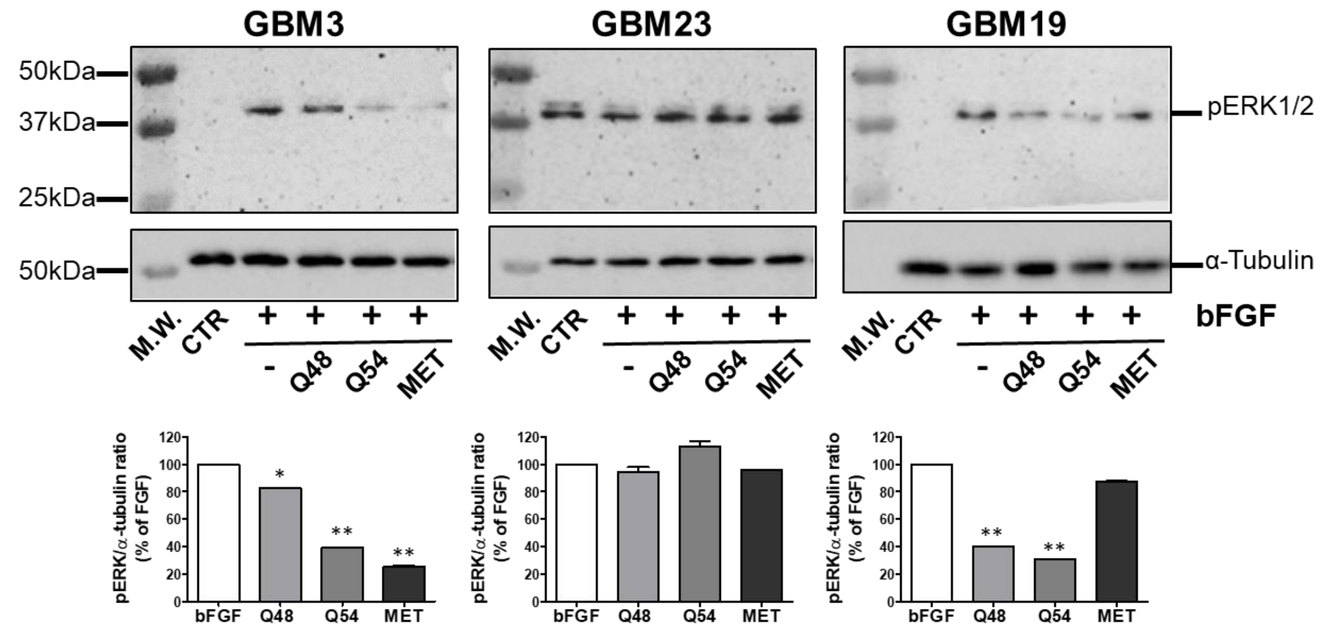

FIGURE S10

Upper panels: Western blot depicting phospho-ERK1/2 (pERK) levels in GSC cultures in control conditions (CTR), after 5 min stimulation with bFGF (40ng/ml) and after treatment of bFGF-stimulated cells with with Q48 (100µM), Q54 and Q46 (300µM), and metformin (10mM) for 24h. α-tubulin was used as loading reference. M.W.= molecular weight markers.

Lower panels: densitometric analysis of pERK normalized for α-tubulin levels, * = p< 0.05; ** =p<0.01.

A

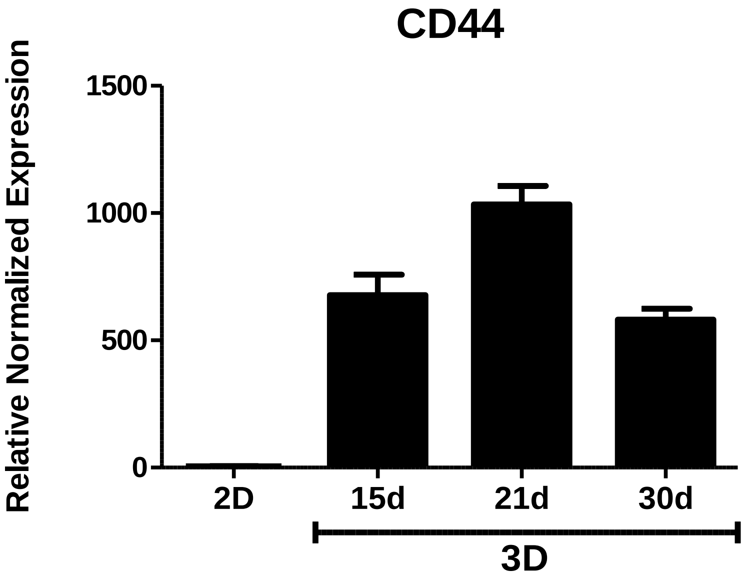

B

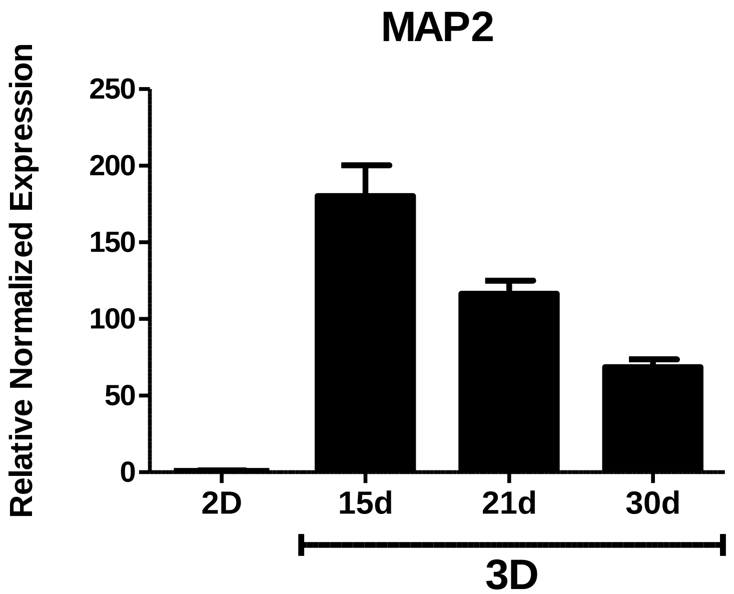

FIGURE S11

Comparison of CD44 (A) and MAP2 (B) mRNA expression in GBM3 GSCs cultivated as 2D monolayer or grown as 3D organoids, for 15, 21, and 30 days. Data are obtained by quantitative RT-PCR experiments.

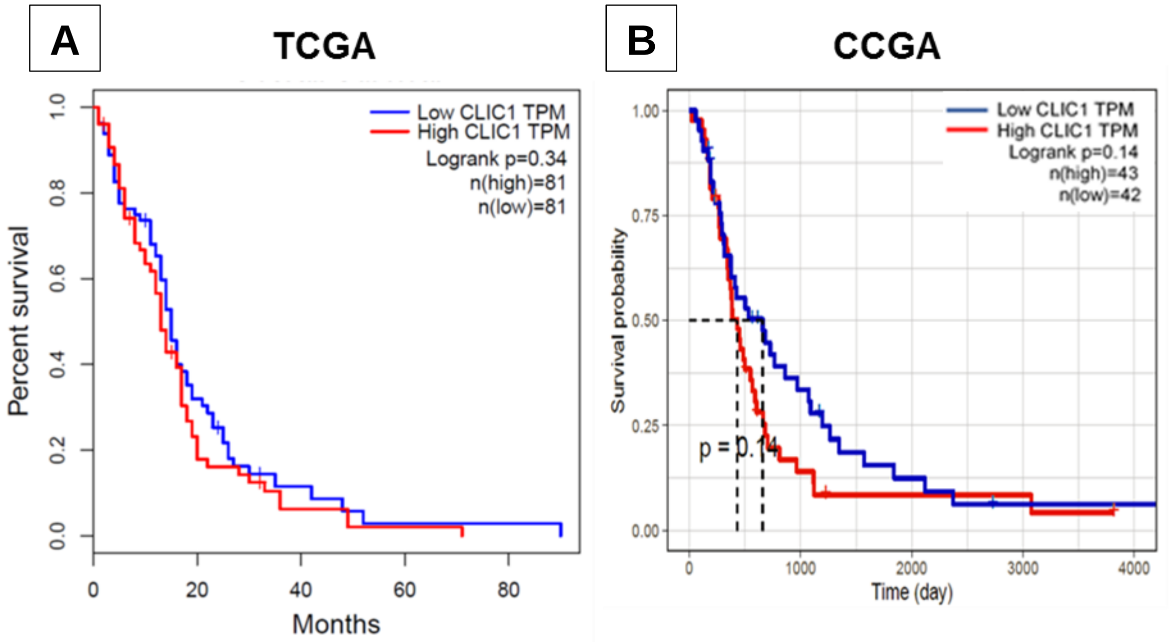

FIGURE S12

Kaplan-Meier analysis of the relationship between overall survival and CLIC1 expression. The statistical difference between the curves is measured by log-rank test.

The prognostic effect of CLIC1 mRNA level in GBM according to The Cancer Genome Atlas (TGCA) databasea nalyzed using the GEPIA (Gene Expression Profiling Interactive Analysis database) software (**A**) and Chinese Glioma Genome Atlas (CGCA) **(B**). TPM: transcripts of per million; n(high): samples with expression level higher than the median of TPM; n(low): samples with expression level lower than the median of TPM.

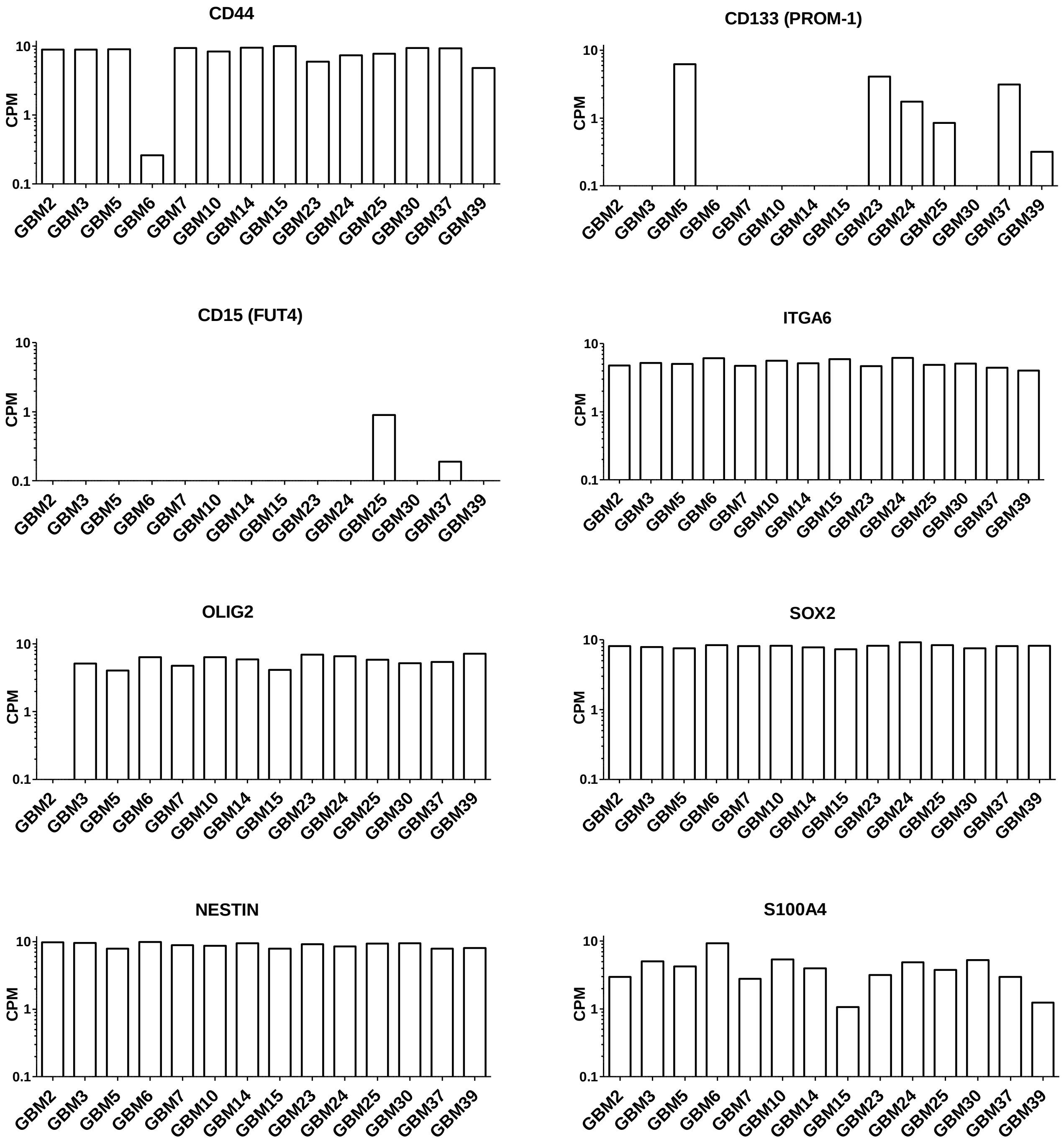

FIGURE S13

Expression stem cell-related markers (CD44, CD133, CD15, integrin-α6 ITGA6, Olig2, SOX2, S100A4, and nestin) in GSCs isolated from 14 human GBMs, evaluated by RNA-seq, and expressed as counts per million reads mapped (CPM). RNA-seq have been data deposited at NCBI Geo data set (see Figure S2 for the relative codes). Low CLIC1-expressing cells (GBM39) do not display differences as compared to high expressing GBMs.

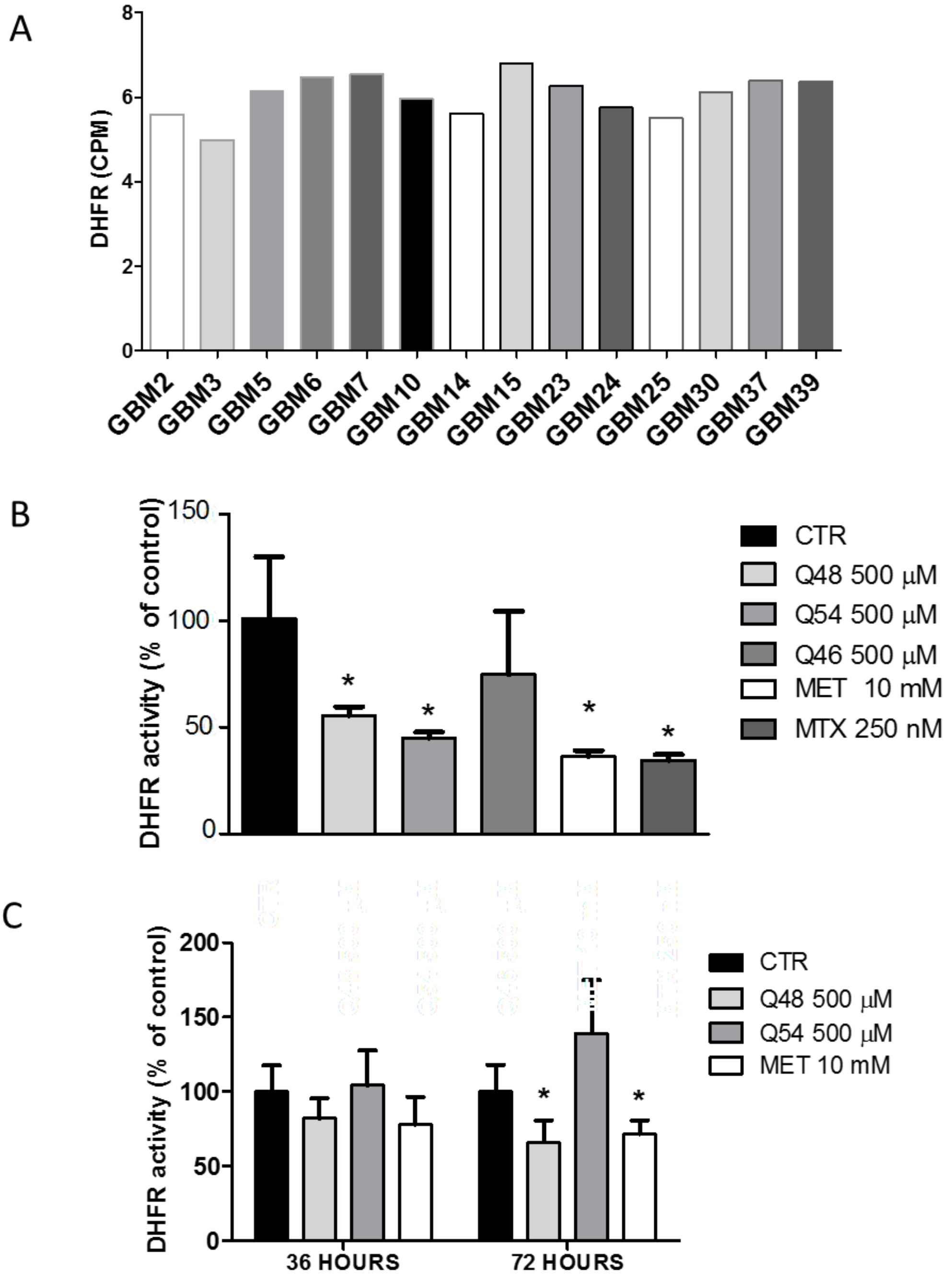

FIGURE S14

A) Dihydrofolate reductase (DHFR) expression in 14 human GSC cultures evaluated by RNA-seq, and expressed as counts per million reads mapped (CPM). Comparable expression was detected in cells derived from all the GBM analyzed. RNA-seq have been data deposited at NCBI Geo data set (see Figure S2 for the relative codes).

B) Effect of Q48, Q54, Q46 and metformin (MET) on DHFR activity, incubating the compounds with purified enzyme. Methotrexate (MTX) was used as positive control. Q48, Q54, and MET, but not Q46, inhibited DHFR activity with an efficacy comparable to MTX. * $p < 0.05$.

C) Effect of Q48, Q54, and metformin, on DHFR activity in living cells. Q48 and metformin, but not Q54, caused a moderate inhibition of enzyme activity in a time-dependent manner. * $p < 0.05$.

SUPPLEMENTARY TABLES

Table S1:

Patients' and tumors' characteristics

Code	Sex	Age (yrs)	WHO grade	Molecular subtype	IDH and 1p/19q co-deletional status	NOD/SCID mice survival time (days)
**GBM 3**	M	48	IV	Neural		120
**GBM 23**	F	70	IV (primary multicentric)	Neural		100
**GBM 19**	F	41	IV (secondary to oligondoglioma)	Mesenchymal		100
**GBM 5**	M	67	IV (primary)	Neural		55
**GBM 10**	F	70	IV			180
**GBM 37**	F	73	IV			150
**GBM 39**	M	52	IV			100
**GBM 44**	F	69	IV		Wt / non-codeleted	n.d.
**GBM 50**	M	71	III		Wt / non-codeleted	n.d.

n. d. = not determined

Table S2:

Elemental analysis of biguanide derivatives

		Calculated %			Found %		
**Compound #**	**Formula**	**C**	**H**	**N**	**C**	**H**	**N**
**Q48**	$C_9H_{13}N_5O$ HCl	44.36	5.79	28.74	44.31	5.65	28.41
**Q49**	$C_9H_{13}N_5$ HCl	47.48	6.20	30.76	47.19	6.03	30.57
**Q50**	$C_9H_{10}F_3N_5$ HCl	38.12	3.94	24.86	38.03	3.88	24.71
**Q51**	$C_{11}H_{15}N_5$ HCl	52.07	6.36	27.60	51.93	6.16	27.87
**Q52**	$C_{11}H_{14}ClN_5$ HCl	45.85	5.25	24.30	45.64	5.00	23.92
**Q53**	$C_{11}H_{14}ClN_5$ HCl	45.85	5.25	24.30	46.02,	5.17	23.90
**Q54**	$C_{12}H_{17}N_5O$ HCl	50.79	6.39	24.68	50.36	6.23	24.45

Q42 (1-(4-chlorophenyl) -biguanide hydrochloride) and Q46 (1-phenylbiguanide hydrochloride) are commercially available and have been obtained by Sigma-Aldrich (purity 97% and 98%, respectively).

Table S3:

Acidity constant (pKa) and distribution constant (LogD) at pH 7 of the novel biguanide derivatives and reference drugs (metformin and cycloguanil)

Compound	pKa*	LogD (pH 7.0)*
Metformin	*12.27*	*-3.25*
**Q46**	11.19	-0.68
**Q42**	11.39	0.09
**Q48**	11.61	-0.89
**Q49**	11.67	-0.28
**Q50**	11.40	0.50
Cycloguanil	*9.27*	*-0.87*
**Q51**	9.77	-1.64
**Q52**	8.96	-0.73
**Q53**	9.20	-0.83
**Q54**	10.4	-1.81

*predicted pKa and LogD values using the Advanced Chemistry Development (ACD/Labs) Software V11.02

Table S4:

Statistical analysis of the effects of novel biguanide derivatives and metformin on GSCs

[mM]	Q42	Q46	Q48	Q49	Q50	Q51	Q52	Q53	Q54	MET
0.003	n.s.	n.s.	n.s.	n.s.	n.s.	n.s.	n.s.	n.s.	n.s.	-
0.01	n.s.	n.s.	n.s.	n.s.	n.s.	n.s.	n.s.	n.s.	n.s.	-
0.03	**	n.s.	***	n.s.	n.s.	*	n.s.	*	*	-
0.1	***	n.s.	***	n.s.	n.s.	*	n.s.	**	**	n.s.
0.3	***	n.s.	***	**	*	**	n.s.	***	***	n.s.
1	-	**	***	***	***	***	*	***	***	n.s.
3	-	***	***	***	***	***	***	***	***	**
10	-	-	-	-	-	-	-	-	-	***
30	-	-	-	-	-	-	-	-	-	***

*p≤0.05; **p≤0.01; ***p≤0.001; n. s. = not significant

(One-way ANOVA, Dunnett's post-test)

Table S5:

Statistical analysis of the effects of novel biguanide derivatives and metformin on differentiated non-stem GBM cells

[mM]	Q42	Q46	Q48	Q49	Q50	Q51	Q52	Q53	Q54	MET
0.003	-	-	n.s.	n.s.	n.s.	n.s.	n.s.	n.s.	n.s.	-
0.01	n.s.	-	n.s.	n.s.	n.s.	n.s.	n.s.	n.s.	n.s.	-
0.03	n.s.	n.s.	n.s.	n.s.	n.s.	n.s.	n.s.	n.s.	n.s.	-
0.1	n.s.	n.s.	n.s.	n.s.	n.s.	n.s.	n.s.	n.s.	n.s.	-
0.3	*	n.s.	n.s.	n.s.	**	n.s.	*	n.s.	n.s.	-
1	*	*	n.s.	**	***	*	***	n.s.	n.s.	n.s.
3	**	**	***	***	***	***	***	*	*	n.s.
10	-	-	-	-	-	-	-	-	-	n.s.
30	-	-	-	-	-	-	-	-	-	*

*p≤0.05; **p≤0.01; ***p≤0.001; n. s. = not significant

(One-way ANOVA, Dunnett’s post-test)

Table S6:

Statistical analysis of the effects of novel biguanide derivatives and metformin on ucMSCs

[mM]	Q42	Q46	Q48	Q49	Q50	Q51	Q54	MET
0.003	*	n.s.	n.s.	n.s.	n.s.	n.s.	n.s.	-
0.01	*	n.s.	n.s.	n.s.	n.s.	n.s.	n.s.	-
0.03	***	n.s.	n.s.	n.s.	***	n.s.	n.s.	-
0.1	***	n.s.	n.s.	n.s.	***	n.s.	n.s.	-
0.3	***	n.s.	n.s.	n.s.	***	*	*	n.s.
1	***	*	n.s.	**	***	**	***	n.s.
3	***	**	*	***	***	***	***	n.s.
10	-	-	-	-	-	-	-	n.s.
30	-	-	-	-	-	-	-	**

*p≤0.05; **p≤0.01; ***p≤0.001; n. s. = not significant

(One-way ANOVA, Dunnett's post-test)

Table S7:

Statistical analysis of the effects of novel biguanides on low CLIC1-expressing GSCs (GBM39 and GBM44) and the average results from high CLIC1-expressing GSCs (GBM3, 5, 19, 23, and 37).

	GBM39			GBM44			High-expressing CLIC1 GSC		
**[mM]**	**Q48**	**Q54**	**MET**	**Q48**	**Q54**	**MET**	**Q48**	**Q54**	**MET**
**0.03**	n.s.	n.s.	-	n.s.	n.s.	-	n.s.	*	-
**0.1**	n.s.	n.s.	-	n.s.	n.s.	n.s.	***	**	n.s.
**0.3**	n.s.	n.s.	n.s.	n.s.	n.s.	n.s.	***	***	n.s.
**1**	n.s.	n.s.	n.s.	n.s.	n.s.	n.s.	***	***	n.s.
**3**	n.s.	n.s.	n.s.	n.s.	*	n.s.	***	***	**
**10**	-	-	n.s.	-	-	n.s.	-	-	***
**30**	-	-	**	-	-	**	-	-	***

*p≤0.05; **p≤0.01; ***p≤0.001; n. s. = not significant

(One-way ANOVA, Dunnett's post-test)
